# Multiscale Modeling of Electronic Spectra Including Nuclear Quantum Effects

**DOI:** 10.1021/acs.jctc.1c00531

**Published:** 2021-09-28

**Authors:** Péter P. Fehér, Ádám Madarász, András Stirling

**Affiliations:** † Institute of Organic Chemistry, Research Centre for Natural Sciences, Magyar tudósok krt. 2, 1117 Budapest, Hungary; ‡ Department of Chemistry, Eszterházy Károly University, Leányka u. 6, 3300 Eger, Hungary

## Abstract

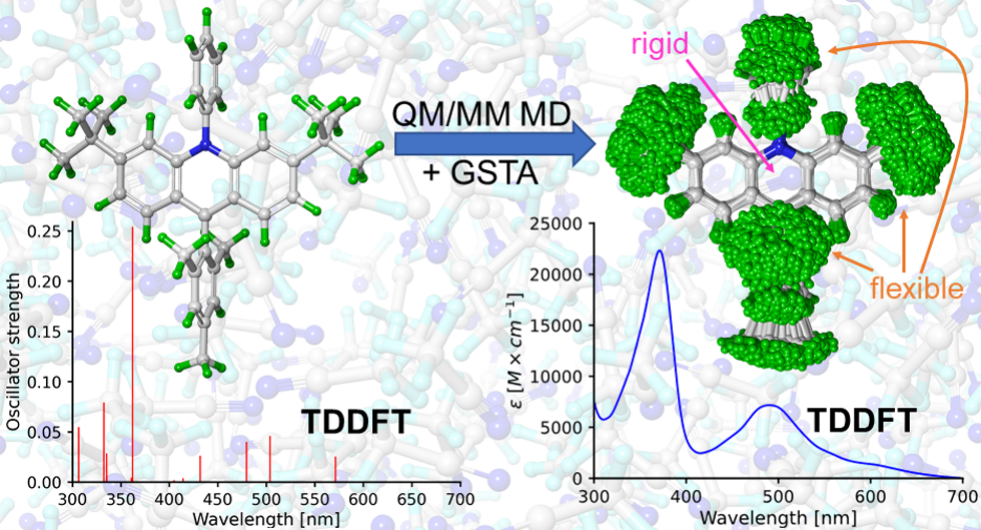

Theoretical prediction of electronic absorption spectra without input from experiments is no easy feat, as it requires addressing all of the factors that affect line shapes. In practice, however, the methodologies are limited to treat these ingredients only to a certain extent. Here, we present a multiscale protocol that addresses the temperature, solvent, and nuclear quantum effects as well as anharmonicity and the reconstruction of the final spectra from individual transitions. First, quantum mechanics/molecular mechanics (QM/MM) molecular dynamics is conducted to obtain trajectories of solute–solvent configurations, from which the corresponding quantum-corrected ensembles are generated through the generalized smoothed trajectory analysis (GSTA). The optical spectra of the ensembles are then produced by calculating vertical transitions using time-dependent density-functional theory (TDDFT) with implicit solvation. To obtain the final spectral shapes, the stick spectra from TDDFT are convoluted with Gaussian kernels where the half-widths are determined by a statistically motivated strategy. We have tested our method by calculating the UV–vis spectra of a recently discovered acridine photocatalyst in two redox states. Vibronic progressions and broadenings due to the finite lifetime of the excited states are not included in the methodology yet. Nuclear quantization affects the relative peak intensities and widths, which is necessary to reproduce the experimental spectrum. We have also found that using only the optimized geometry of each molecule works surprisingly well if a proper empirical broadening factor is applied. This is explained by the rigidity of the conjugated chromophore moieties of the selected molecules, which are mainly responsible for the excitations in the spectra. In contrast, we have also shown that other parts of the molecules are flexible enough to feature anharmonicities that impair the use of other techniques such as Wigner sampling.

## I Introduction

Photocatalysts employed under homogeneous catalytic conditions are molecules that harvest the energy of visible light to facilitate new transformations and new synthetic routes that may yield otherwise inaccessible scaffolds and molecules.^1^ The typical photocatalytic scenario is that upon interaction with light the photocatalyst molecules reach accessible excited states and then they quench rapidly to the lowest available excited state within the same spin manifold (e.g., a singlet molecule will be in the S_1_ state). Depending on the circumstances, the further interconversions define the subsequent feasible catalytic processes such as reaching a T_1_ (first triplet) state and then engaging in various electron transfers to trigger a photoredox transformation. The molecules typically employed in photocatalysis absorb visible light, a feature that has a significant advantage: they can be selectively excited in the visible region, whereas typical organic substrates and solvents absorb in the UV region. This selectivity can be achieved by inserting and tuning chromophore groups, such as delocalized π-systems. The electronic properties of photocatalysts can be explored by measuring or calculating their electronic (UV–vis) spectra. In this regard, calculations can be very useful because they provide a large amount of information not directly available from experiments, such as the assignation of bands to transitions between electronic states or the identification of dark states. Calculations can also help to understand how structural and electronic modifications introduced to photocatalysts affect the excitations, therefore facilitating the design of new photocatalysts.

These computations, however, have to address a number of challenges. To begin with, the description of the electronic structure in both ground and excited states requires sufficiently accurate methods.^2^ In practice, time-dependent density-functional theory (TDDFT) employing functionals with exact exchange contribution is usually adequate;^3^ but, more accurate, wavefunction-based methods should be considered for higher accuracies. Another issue is to capture effects arising from fluctuations induced by the environment such as temperature and solvent. In fact, routine calculations can only provide a crude approximation to these as they use a single configuration to obtain the excitation spectrum.^4−6^ For benchmarking purposes, however, this strategy is still employed to evaluate the performance of exchange–correlation functionals. The better way to account for the environment is through the nuclear ensemble approach, where the feasible nuclear configurations are sampled and then the final simulated spectrum is obtained as the sum of the individual spectra of the configurations.^5−11^ Sampling is usually done using molecular dynamics (MD) or Monte–Carlo (MC) simulations. Both of these can sample anharmonic regions of the ground-state potential energy surface (PES), which is essential for molecules with an inherently flexible nature.

Equally important challenge is to include the effects of the environment on the optical spectra at both stages of the simulations: for the electronic structure calculations and for the sampling of the nuclear configurations.^4,13^ The environment has an enormous impact on the optical spectra because it influences the ground and excited states and it can change drastically the distribution of nuclear configurations. A number of considerations are due in this respect as well. The most accurate models include steric and electrostatic interactions between the solute and the solvent environment at both short and long ranges, i.e., via explicit solvent molecules and accurate quantum mechanical treatment. The cost of calculations can be reduced by introducing further approximations in the treatment of solvent molecules, such as simplified electronic structure description or employing suitable force fields (quantum mechanics/molecular mechanics (QM/MM), multiscale approaches). An enormous cost reduction can be achieved with implicit solvent models where the solvent degrees of freedom are excluded from the calculations but the electrostatic interactions are still included in the model.

It can also be crucial to take into account the quantum nature of nuclear motions when generating the nuclear ensemble.^6,10,12,14,15^ This can be done in different ways. The proper ground-state nuclear density distribution can be obtained from path-integral MD simulations although it can be very costly for larger systems.^16^ Then, the vertical excitations are calculated for the configurations of the sample and summed up for the spectrum (see, e.g., ref (15)). Another option is to evaluate the Franck–Condon (FC) overlaps between the vibrational levels of the ground and excited states.^17^ In practice, this method is used within the harmonic approximation and accounts for the vibronic fine structure of the spectra. Within the harmonic approximation, the Wigner sampling^5,18−24^ and also the LQ2 method^25^ are also a suitable choice to represent the nuclear quantum distribution. These methods were shown to approximate well the envelope of the exact absorption spectra but they cannot reproduce the vibrational fine structure directly. Also, the Wigner method fails for highly flexible molecules because the harmonic approximation cannot be applied.^26^ González et al. proposed two new sampling protocols to simulate the absorption spectra called “local temperature adjustment” and “individual QM/MM-based relaxation”.^26^ In these approaches, the chromophore is thermostated at an elevated temperature while the solvent remains at room temperature to introduce zero-point energy. Although all nuclei are treated classically, the spectral lines are broadened reasonably well with these protocols.

Recently, a new method has been introduced to obtain quantum-corrected trajectories and in turn quantum-corrected structural properties and state functions such as heat capacities.^27^ The method is called generalized smoothed trajectory analysis (GSTA) and is based on the idea that the quantum effects can be recovered from a classical trajectory by convoluting the classical coordinates with an appropriate kernel function derived from the quantum harmonic partition function. It has been shown that quantum effects can be reproduced in a very efficient and cost-effective way. In particular, the results are of comparable accuracy to those obtained from path-integral calculations but at a fraction of the costs.

**Figure 1:**
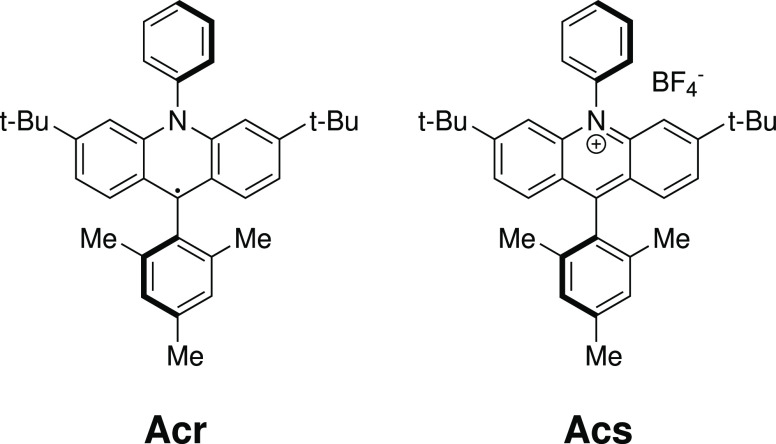
Photocatalyst molecules selected for this study. **Acr**: 3,6-di-*tert*-butyl-9-mesityl-10-phenyl-9,10-dihydro-9λ^3^-acridine; **Acs**: 3,6-di-*tert*-butyl-9-mesityl-10-phenylacridin-10-ium tetrafluoroborate.

Our current interest in computational modeling of photocatalysis led us to a recently discovered photocatalyst radical and its ionic counterpart (Figure 1). Both the mesityl-acridinium salt (Mes-Acr^+^BF_4_^–^, **Acs**) and its reduced derivative, the radical Mes-Acr^•^ (**Acr**), are photocatalytically active; moreover, the radical, when excited, has a reduction potential equivalent to that of the element Li, i.e., it is a very powerful organic reductant.^28^

In this study, we simulate the optical spectra of these photocatalysts taking into account the experimental conditions (room temperature and acetonitrile (ACN) as a solvent). The aim of this study is to devise and test a protocol that is suitable to determine electronic absorption spectra without any experimental input for the simulated line shapes. To this end, we have designed a multiscale computational strategy: first QM/MM MD simulations have been performed to obtain a sufficiently large set of configurations of the catalyst molecules in explicit solvent (acetonitrile). Then, the GSTA method has been used to obtain a quantum-corrected ensemble of solvated photocatalyst molecules. Finally, the optical spectra of the molecules have been calculated with TDDFT for both the uncorrected and corrected configurations. In this step, only configurations of the catalyst molecule taken from the QM/MM trajectories (original or quantum-corrected) have been considered with implicit solvation employing the parameters of the same solvent (acetonitrile). Similar multiscale approaches have already been employed to obtain absorption spectra.^2,12,29−31^ Particular attention has been paid to the reconstruction of the final classical and quantum-corrected spectra from the individual peaks because we want to separate the real physical effects of the spectral broadening from the pure mathematical operations such as the smoothing of spikelike spectra to obtain a continuous function from a limited number of data points.

## II Methods

The absorption spectrum of a single molecule in atomic units can be written^9,32^ as
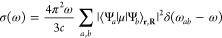
1where ω is the angular frequency, ω*_ab_* = *E_a_* – *E_b_*, *E_a_*, and *E_b_* are the energies of the initial and final molecular states *a* and *b*, respectively (note that ℏ = 1 in atomic units and therefore it is omitted from the equations), *c* is the speed of light, Ψ*_a_* and Ψ*_b_* are the full, molecular wavefunctions of states *a* and *b*, and μ is the electric dipole moment. The ket indexes **r** and **R** are the integration coordinates for the electrons and nuclei, respectively.

Assuming the validity of the Born–Oppenheimer approximation (BOA), the wavefunction of an arbitrary state can be written as the product of the nuclear (θ(**R**)) and electronic (ϕ(**r**; **R**)) wavefunctions: Ψ_*n*_ = θ_*k*ν_(**R**)ϕ_*k*_(**r**; **R**), where **r** denotes the electronic coordinates and **R** denotes the nuclear coordinates, which appear as parameters for the electronic wavefunctions. *k* indexes the electronic states and ν indexes the rovibrational states. The eigenvalues (*E*_*k*_(**R**)) corresponding to ϕ_*k*_(**r**; **R**)-s define the potential energy surfaces (PESs). We also assume that the vibrational motions can be fully separated from the rotational motions; hence, the rotational wavefunctions do not appear in the equations and θ_*i*ν_(**R**) from now on denotes the vibrational state number ν of the electronic state *i*. The electric dipole moment can also be written as the sum of nuclear and electronic dipoles: μ = μ_*n*_(**R**) + μ_*e*_(**r**). Due to the orthogonality of the electronic states, the terms ⟨Ψ_*a*_|μ|Ψ_*b*_⟩ become, in BOA, ⟨θ_*i*ν_(**R**)ϕ_*i*_(**r**;**R**)|μ|θ_*j*ν′_(**R**)ϕ_*j*_(**r**;**R**)⟩_**rR**_, = ⟨θ_*i*ν_(**R**)|μ_*ij*_(**R**)|θ_*j*ν′_(**R**)⟩_**R**_, where μ_*ij*_(**R**) = ⟨ϕ_*i*_(**r**;**R**)|μ_*e*_(**r**)|ϕ_*j*_(**r**; **R**)⟩_**r**_ is the electronic transition dipole. The absorption spectrum of a single molecule is then written as
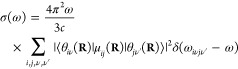
2where ω_*i*ν*j*ν′_ is the energy difference between the initial and final vibronic states *i*ν and *j*ν′.

In practice, we have an equilibrium ensemble of absorbing molecules where their relative concentrations are characterized by their probability distribution ρ_*i*ν_. After excitation, they can assume the possible vibronic states. Therefore, σ(ω) can be written as
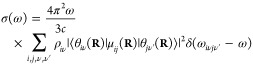
3In a typical experiment, at ambient temperature, molecules in their ground electronic state contribute predominantly to the absorption spectrum; therefore, the expression for the absorption cross section becomes
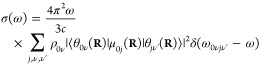
4The term ω_0ν*j*_*ν′* in the Dirac-δ can be approximated as ω_0ν*j*ν′_ ≈ ω_0*j*_(**R**), i.e., with the vertical excitation energy from the ground state of configuration **R** to the turning point of the classical harmonic oscillator on the *j*th PES (reflection approximation^33−35^). This implies that no vibronic fine structure is captured. Because of the **R**-dependence, the Dirac-δ is now transferred into the integral and the absorption spectrum takes the form
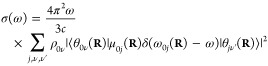
5Further simplification can be done using the completeness relation of the vibrational eigenstates: ∑_ν′_|θ_*k*ν′_⟩⟨θ_*k*ν′_| = 1; hence
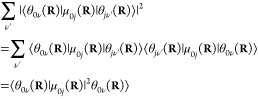
The absorption cross section now takes the form
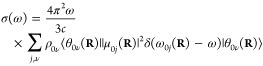
6Since the δ-function selects the ω_0*j*_(**R**) values, ω can be taken inside the integral. We can then rewrite the expression by introducing the oscillator strength 
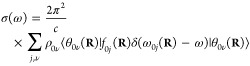
7Writing explicitly the integration, we can notice that the summation can be expressed in a practical form
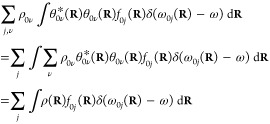
where ρ(**R**) = ∑_ν_ρ_0ν_θ_0ν_^*^(**R**)θ_0ν_(**R**) is the probability distribution of configuration **R** on the electronic ground-state PES. The absorption cross section can now be written as
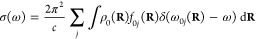
8We found that the absorption spectrum can be calculated to a good approximation from the ground-state distribution of the molecules and from the vertical excitation energies. This approach is called the nuclear ensemble method and has been employed successfully to reproduce absorption spectra.^5−7,9−11^ It is important to note that the configurational integral is a faithful representation of the model employed to compute the absorption spectra because it includes not only the target molecule but also the effect of the environment, such as solvents. In addition, the temperature dependence of the absorption spectra is also captured by the distribution function. Note, however, that within this approximation the vibrational structure of the excited states is lost. In contrast, depending on how we sample the configurational integral, the ground-state quantized vibrational structure can be recovered in the calculations.

In practice, the sampling of ρ_0_(**R**) can be done by MD or MC methods or using Wigner sampling. In the case of *N* samples representing ρ_0_(**R**), the practical form of eq 8 is
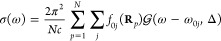
9where  is a Gaussian-type broadening function centered at ω_0*j*_ with a standard deviation of Δ to smear the spikes of the δ function. It is important to notice that eq 9 specifies the main issues of the simulations:

(i) proper sampling of the ground-state nuclear configurations;
(ii) selection of the proper smearing function to smooth the spike spectra;
(iii) the electronic structure method to obtain sufficiently accurate excitation energies and oscillator strengths.

### Sampling

Configurational sampling can be performed in a number of ways; however, all of them follow one of the two fundamental strategies. The faster approach is to start from an optimized geometry and the corresponding Hessian; then, displace the nuclei along the normal modes to obtain an ensemble that conforms to an analytical probability density function.^8^ The most common function is the harmonic Wigner distribution because it accounts for the nuclear quantum effects.^18^ The other main approach is to perform molecular dynamics (MD) calculations, where quantum effects can be included in various ways. The advantage of MD sampling is that it can be applied in cases where the harmonic approximation fails (e.g., due to the presence of low-frequency modes). As we show later, the harmonic Wigner distribution yields highly dubious results even for our test molecules that feature only a limited amount of low-frequency contributions. Therefore, in the present study, we sampled the configurational space with QM/MM MD.

The MD trajectories were generated in a periodically repeated cube of side length 30 Å using the CP2K program package.^36^ The substrate **Acr** was solvated by 298 acetonitrile (ACN) solvent molecules, whereas for the substrate **Acs** the simulation box contained 295 ACN molecules. In this way, the densities were slightly higher than that of the bulk ACN. The QM cubic box inside has a dimension of 20 × 20 × 20 Å^3^. The simulations have been done under NVT conditions at 300 K employing separate CSVR thermostats^37^ for the solvent and the solute. The time step was 0.5 fs. We have also verified that this step size is sufficient to ensure energy conservation during simulations by performing NVE calculations on equilibrated systems. The protocol for obtaining trajectories was the following: first, the solutes (**Acr** or **Acs**) were optimized keeping the solution frozen, then the solvent is equilibrated for a few picoseconds (ps) while the solute molecules were kept fixed; then, we performed long NVT equilibrations till the temperature of both the solute and solvent reached and remained around 300 K. In the case of **Acr**, 9 ps was required for the equilibration, whereas, for the salt **Acs**, 5 ps was needed. After equilibration, production runs of 45 and 29 ps have been performed for **Acr** and **Acs**, respectively. The classical trajectories obtained in this way include anharmonicity; however, nuclear quantum effects need to be treated separately. This was done using the GSTA method^27^ for which we give here a short summary. In GSTA, the classical nuclear trajectories (as well as velocities and forces that are however not important for the present study) are convoluted with an appropriate kernel function corresponding to the harmonic oscillator approach to obtain the quantum-corrected structures
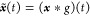
10where ***x***(*t*) is the original (classical) trajectory and ***x̃***(*t*) is the filtered (quantized) trajectory. Convolution is represented by *. The filtering function *g* is defined as
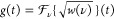
11where  indicates the Fourier transformation in the frequency domain ν, and *w*(ν) is the weighting function that gives the ratio of the energies of the quantum and classical harmonic oscillators
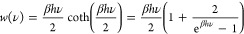
12where coth is the hyperbolic cotangent function, β = (*k*_B_*T*)^−1^, *k*_B_ is the Boltzmann constant, *T* is the temperature, and *h* is the Planck constant. In the present case, the convolution is done by scanning the trajectories with a moving window over 241 frames (120 fs). For the calculations of the absorption spectra, substrate configurations in every 100th frame from both the classical and filtered trajectories were used. The program code used for filtration is available on GitHub.^38^

### Finding the Parameters of the Proper Broadening Function

The spectrum given by eq 8 for an ensemble of configurations can be, in principle, transformed to a histogram with an appropriately chosen bin-width. In practice, however, we prefer to use a sum of Gaussians (eq 9), so the spectrum can be expressed as a smooth function of energy in the following practical form
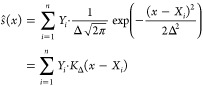
13where *K*_Δ_ is the kernel function of width Δ, *X*_*i*_ is the calculated excitation energy, and *Y*_*i*_ is the corresponding oscillator strength at this energy. The width of the kernel function (Δ) is a parameter with the property of Δ → 0 as *N* → ∞.^9^ However, the selection of Δ in practice is far from obvious. Usually, the bandwidth for the theoretical spectrum is arbitrarily set to afford the best agreement with the experimental spectrum.^34,39^ In contrast, we seek here a statistically motivated strategy that does not require any information about the experimental spectrum and is also able to separate the artificial broadening caused by the kernel functions from the broadening produced by nuclear quantization or the other effects included in the configurational sampling. To this end, we have combined two strategies to find the optimal Δ values for each spectrum. First, with a selected Δ, we can obtain an optimal weighting parameter (*a*(Δ)) for all of the kernels after minimizing an *L*(*a*;Δ) cost function defined as
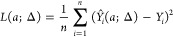
14by considering the mean integrated squared error between the originally calculated oscillator strengths (*Y*_*i*_) and those given by the kernel functions when the parameter *a* is varied
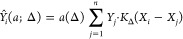
15Then, with the optimal *a*(Δ) in hand, we can calculate another cost function *L*_*cv*_(Δ) corresponding to the leave-one-out cross-validation,^40^ i.e., how well a single *Y*_*i*_ is predicted by the sum of the kernels when the selected data point *X*_*i*_ is not included in the calculation. *L*_*cv*_(Δ) is the sum of the squared differences of the original and predicted absorption cross sections
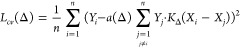
16Minimizing *L*_*cv*_ yields the optimal kernel width Δ, which we use to obtain the absorption spectra. The plots of *L*_*cv*_ vs Δ for all of the six calculated trajectories are shown in Figure S1. As a summary of this section, the *a*(Δ)*ŝ*(*x*) function yields an optimal fit of the oscillator strength vs energy (*Y*_*i*_ vs *X*_*i*_) data points and was determined by a kernel regression technique.

### Electronic Structure Methods

In the QM/MM simulations, the solute photocatalyst molecules have been described by the PBE-D3 DFT functional.^41^ A hybrid Gaussian/plane wave (GPW) basis set scheme has been used where the valence atomic orbitals are expanded on a short-range molecularly optimized DZVP basis set,^42^ whereas the corresponding one-electron densities are expanded over a plane-wave basis set defined by a cutoff of 300 Ry. The effects of atomic cores were described by GTH pseudopotentials.^43^ The CHARMM force field^44^ has been used for the flexible solvent, and the nonbonded parameters for all atoms were also taken from this force field. The method developed by Laino et al.^45^ was used to calculate the electrostatic couplings between the QM and MM parts.

The electronic spectra have been calculated for the ensemble of solute configurations extracted from every 100th snapshot of the trajectories. For these calculations, the Gaussian09 program package has been used.^46^ The ground- and excited-state electronic structures have been obtained by TDDFT using the B3LYP functional.^47^ We have compared our results with those obtained using the M06 functional.^48^ The calculations employed linear response solvation for the vertical absorptions (solvation model based on density (SMD) implicit solvent model of ACN).^49^ The orbitals were expanded on the triple-ζ basis set of Ahlrichs et al., completed with a set of polarization functions (TZVP).^50^ We have considered excitations up to 4.2 eV (corresponding to ca. 300 nm), which in practice required the calculation of 25 states.

When we switch from explicit to implicit solvent representation, a fraction of solvent-induced broadening may be lost because only the ensemble of solute configurations is considered in the spectrum calculations. The origin of this issue is that similar or identical solute configurations in the trajectory can be surrounded by different solvent configurations. When implicit solvation is applied subsequently, this can lead to a loss of broadening because these solute configurations yield identical or very similar spectra.

The primary quantities obtained from the electronic structure calculations are the excitation energies. Hence, the plot of σ(*E*) or σ(ω) is the natural choice to represent the absorption spectra. In contrast, UV–vis measurements typically express the absorption cross section as a function of wavelength λ. Therefore, it seems sensible to convert our results from energy units to wavelength units. To compare line shapes, we also normalized all of the spectra such that the area under the curve in the 300–700 nm interval equals unity. The absolute spectra showing molar extinction coefficients can be found in the Supporting Information (SI).

The approximation used very often in practice is that the electronic transition moment is calculated at a single nuclear geometry, usually at the ground-state equilibrium structure **R**_0_ (single-point approach). In this case, the practical form of eq 9 for the absorption spectrum is reduced to the following
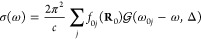
17This approximation is often used in benchmark studies. We have calculated the absorption spectra of the photocatalyst molecules considered in this study employing this approach too and compared the results to the spectra obtained from the trajectories. For this, we have optimized the structures using the PBE-D3 functional taking into account the ACN solvation environment implicitly via the SMD solvation method. Note that we chose PBE-D3 here because the same functional is used to obtain configurations in the QM/MM MD calculations. Then, we have calculated the electronic spectra using TDDFT with the following functionals: B3LYP, M06, PBE,^41^ PBE0,^51^ ω-B97XD,^52^ and CAM-B3LYP.^53^ To achieve the best possible agreement with the experimental spectrum, we have systematically varied the broadening parameter Δ. The optimal value is then assumed to capture the inhomogeneous broadening due to the solvent and temperature^12^ as well as the nuclear quantum effects. This simplification evidently obscures the underlying physics of a given system; however, it yields a reasonably good approximation for the spectrum of **Acr**.

## III Results and Discussion

**Figure 2:**
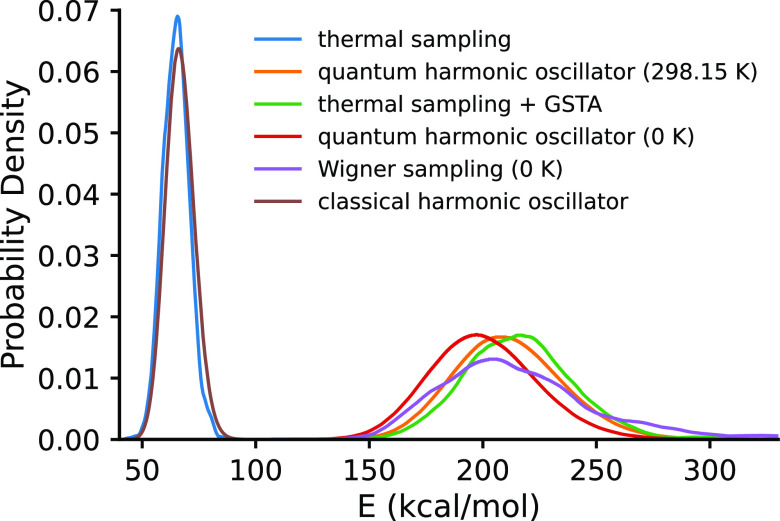
Potential energy distributions with different configurational sampling techniques. The Wigner sampling at 298.15 K is not shown as it produced a large number of fragmented structures (see Figure 3). The energy values are given relative to the Kohn–Sham energy of the optimized **Acr** structure.

**Figure 3:**
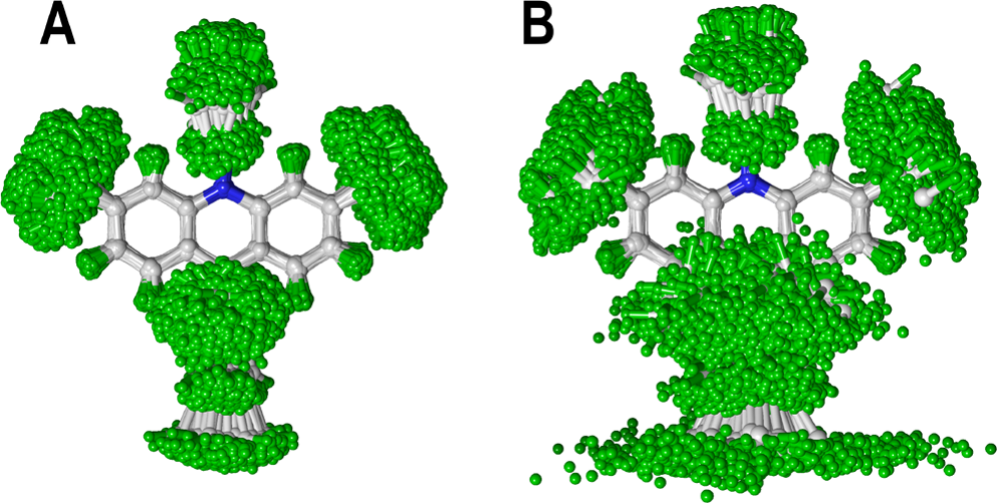
Comparison of the quantized nuclear distributions for **Acr**: (A) 806 superimposed nuclear configurations obtained from GSTA correction; (B) 1000 superimposed nuclear configurations obtained from the Wigner sampling at 298.15 K. Color code: hydrogen, green; nitrogen, blue; carbon, gray.

First, we compare the results of the Wigner and MD samplings. The energy distribution of the different ensembles is shown in Figure 2. The Wigner sampling has been performed using the Newton-X program.^54^ The distribution of the potential energy obtained from thermal sampling is very close to the χ^2^-distribution that can be derived from the classical harmonic model.^55^ The two overlapping curves at 65 kcal/mol show this agreement. When the zero-point energy is taken into account, the distribution maxima are shifted from 65 to 200–220 kcal/mol and the width of the distributions increases from 6 to 20–40 kcal/mol. If the vibrations were perfectly harmonic, then the energy distribution from the Wigner sampling would be identical to the quantum harmonic model. Wigner sampling at 0 K, however, affords structures with significantly higher energies, indicating notable anharmonicity. We also performed the sampling at *T* = 298.15 K and obtained chemically unrealistic, fragmented structures in a surprisingly high ratio. A graphical comparison of the GSTA and Wigner sampling is given in Figure 3. For example, ca. 60% of the structures feature C–H distances larger than 2 Å. In contrast, the combination of MD and GSTA methods yields a distribution very similar to that of the quantum harmonic model at room temperature. This similarity indicates that most vibrations are harmonic and the thermal sampling with GSTA works sufficiently, although there are a number of low-frequency modes (e.g., methyl rotation) that impairs the application of Wigner sampling.

**Figure 4:**
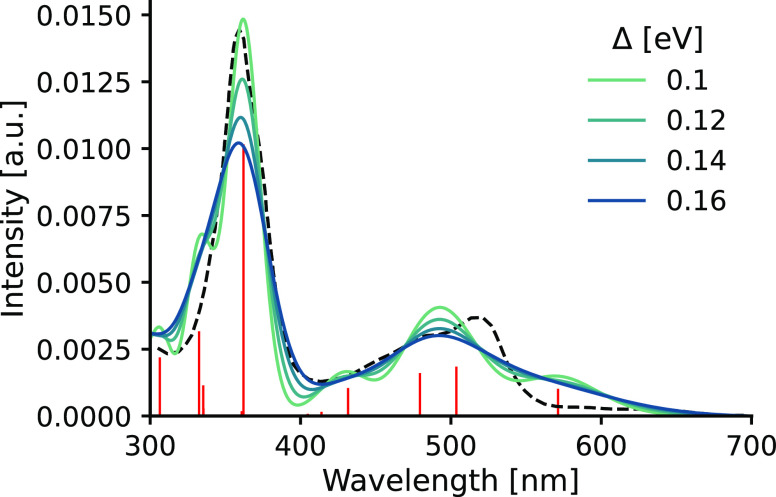
Comparison of the spectra obtained from the single-point convolution approach for **Acr** in ACN solution employing different Gaussian widths for eq 17 with the experimental spectrum (dashed). The stick spectrum in red represents the calculated excitations without Gaussian broadening. Lower Δ values are shown in Figure S2.

To obtain reliable electronic spectra for the configurations of **Acr** and **Acs** obtained from QM/MM simulations, we have tested six exchange–correlation functionals that are commonly used for excited-state calculations. This test is done within the framework of the single-point approximation. For a meaningful comparison with the experiment, however, we first need to determine the optimal Gaussian broadening parameter (see eq 17) to apply to the stick spectra provided by TDDFT. Figure 4 shows the evolution of the calculated spectra of **Acr** as a function of the broadening parameter and also includes the experimental spectrum extracted from ref (28). We use the B3LYP functional for TDDFT together with linear response SMD solvation that accounts for the nonequilibrium environment of acetonitrile in the vertical excited state. This functional selection is based on our preliminary exploratory functional tests.

We can make a couple of interesting observations. The raw stick spectrum aligns quite well with the experiment, especially at the 360 nm peak. By applying the Gaussian broadening and gradually increasing the Δ parameter, the close-lying excitations start to merge and the calculated spectrum develops into the shape resembling the measured spectrum from Δ > 0.12 eV. A wider range of Δ values is shown in Figure S2. It can be seen that, eventually, the spectrum becomes featureless as all of the peaks are merged into a single band that has a maximum of around 340 nm and a very long tail region. Note that increasing Δ also provides a blueshift that is clearly visible at the 360 nm peak. It is due to the presence of additional excitations below 300 nm. Therefore, we conclude that Δ has an optimal value of 0.14 eV when approximating the absorption spectra of **Acr** with the single-point approach. This result is in line with the values (0.2–0.4 eV) often recommended to reproduce experimental line shapes from single-point calculations.^7,39^ It is important to note, however, that this empirical strategy to obtain optimal bandwidths can be followed only if experimental measurements are available.

**Figure 5:**
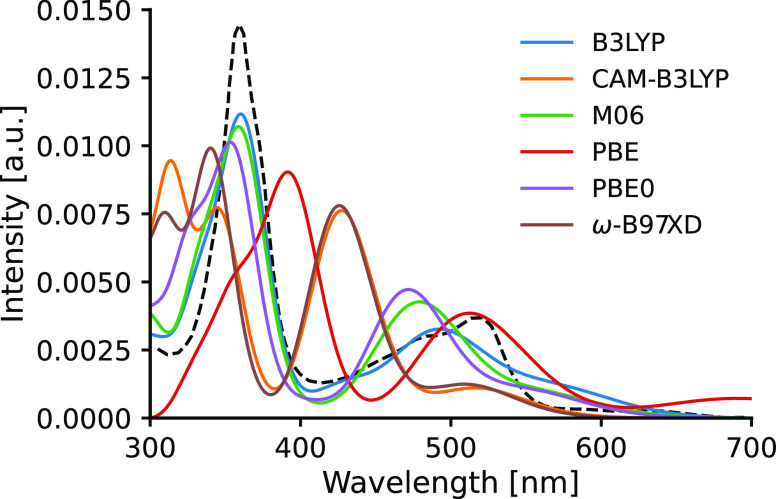
Comparison of the performance of selected functionals for **Acr**. The dashed line corresponds to the experimental spectrum.

**Figure 6:**
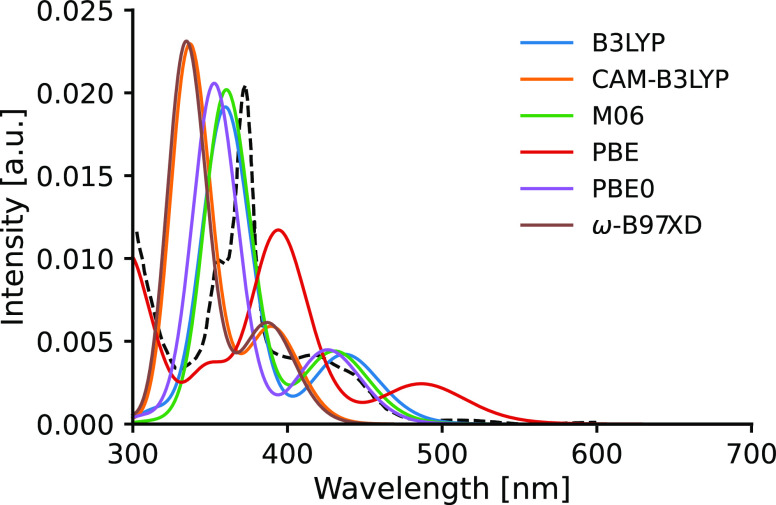
Comparison of the performance of selected functionals for **Acs**. The dashed line corresponds to the experimental spectrum.

We can now compare the single-point spectra calculated with the selected functionals with the experiment using an optimal Gaussian width of 0.14 eV. This comparison is shown in Figure 5 for **Acr** and in Figure 6 for **Acs**. We can see in Figure 5 that the spectra obtained with functionals B3LYP and M06 show very good agreement with the experiment, while PBE0 offers similar accuracy with a 10–20 nm blueshift. It is also apparent that the spectra obtained with range-separated functionals ω-B97XD and CAM-B3LYP are very similar to each other, albeit with some alterations in intensities in the high energy (i.e., low wavelength) region. However, they feature a consistent ca. 40 nm blueshift with respect to the experiment and an increased intensity for the lower-energy bands. In contrast, the PBE functional (which is the only functional considered here without any exact Hartree–Fock exchange) predicts an absorption spectrum redshifted by ∼40 nm, indicating considerable underestimation of the excitation energies. The computed spectra of **Acs** show the same patterns seen for **Acr**: B3LYP, M06, and PBE0 provide almost identical spectral shapes and a very good agreement with the experimental 375 and 420 nm peaks, while ω-B97XD and CAM-B3LYP yield identical spectra blueshifted by 20–40 nm and the PBE functional again underestimates the excitations by 40–50 nm. Based on these results, we use the B3LYP and M06 functionals to calculate the spectra in the ensemble average. Note that it is known that the reliability of functionals in TDDFT calculations is not uniform over a wider range of molecule types;^56^ therefore, our observations regarding the performance of the functionals are valid only for these photocatalysts. In this context, it is also important to mention that the absorption spectrum calculations for **Acr** using the single-point approach are also given by Nicewicz et al. in ref (28). They employed a screened range-separated hybrid functional - polarizable continuum model (PCM) method,^57^ where the functional is ωPBEh.^58^ After comparison with the experiment and B3LYP results, the performance of this range-separated functional was deemed superior as it reproduced the intensive experimental transition at 2.31 eV (537 nm) as well as the dark state at 2.76 eV (449 nm) with negligible error. Since dark states are typically associated with charge-transfer excitations, this result is expected as range-separated functionals capture long-range effects more efficiently than pure and hybrid functionals. However, the final shape of each spectrum is built up from multiple excitations from which the ones with low oscillator strengths yield low contributions. Therefore, the accurately predicted dark states from range-separated functionals as well as the low-energy spurious transitions from global hybrids^59^ are less apparent in the spectrum. This provides a noticeable advantage for the global hybrids in Figure 5.

**Figure 7:**
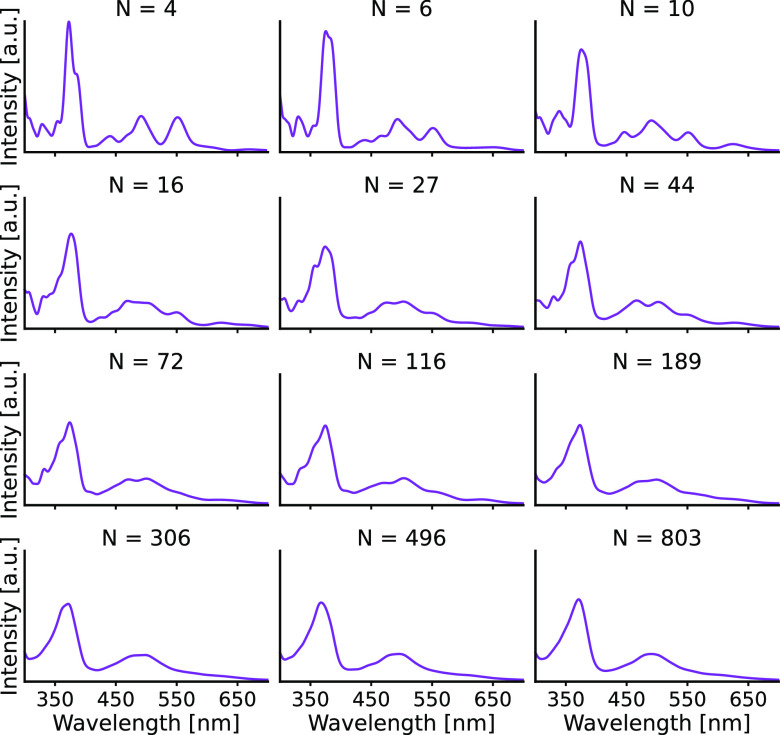
Convergence of the calculated absorption spectra as a function of logarithmically increasing number (4, 6, 10, 16, 27, 44, 72, 116, 189, 306, 496, 803) of frames.

The next step in our study is to ensure that the QM/MM samplings are converged. To this end, we inspected how the calculated absorption spectra vary as a function of snapshots. The snapshots are taken from the QM/MM trajectories at every 50 fs. Figure 7 displays the absorption spectra for ensembles of an increasing number of snapshots. All spectra have been obtained by employing the optimal Gaussian width (0.045 eV) for eq 16. It can be seen that for around 500 snapshots the ensemble-averaged absorption spectrum is already converged. To verify that the converged spectrum represents uncorrelated MD snapshots, we have calculated ensemble spectra from 400 randomly selected frames 12 times and compared them (Figure S3). Although we did not set any criterion for the comparison, a simple inspection of the spectra shows that they are nearly identical. Therefore, we conclude that the absorption spectrum is properly converged.

**Figure 8:**
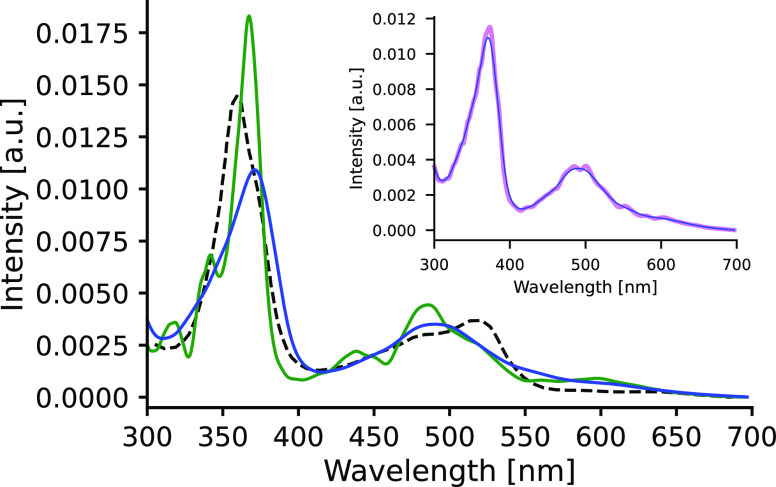
Comparison of experimental and theoretical electronic spectra of **Acr** in ACN solution. Color code: green, spectrum from classical simulation; blue, spectrum from the quantized trajectory; dashed black, experimental curve. Inset: comparison of the GSTA-filtered spectra employing different kernel widths; violet: 0.022 eV; blue: 0.045 eV.

Figure 8 displays the spectra obtained for the **Acr** radical both with and without nuclear quantum effects. The broadening of the bands in the classical spectrum is due to the presence of various conformations and to the changes in the electronic spectra of these conformations induced by solvent fluctuations (inhomogeneous broadening).^5,12,60^ On top of these, the quantum-corrected spectrum includes the additional broadening effect of zero-point energy, which is clearly discernible in Figure 8. In fact, this is the expected effect of quantization, as the energy of the zero-point vibrational motion (*E*_ZPV_ = *h*ν/2) is usually higher than the average classical energy (*E*_th_ = 1/β) available for a harmonic oscillator at ambient temperature, especially for higher-energy vibrations. This implies that the ratio of the amplitudes obtained from quantized and thermal samplings for a given internal degree of freedom can be quite large. Within the harmonic approximation, a lower estimate is the ratio , which can be as high as 2–3 for X–H (X = C, N, O) stretchings.^6^ This is in line with eq 12, which is used to generate the quantized nuclear ensembles. It shows that the energy of the real, quantized vibration is always higher than that of the classical vibration, and, at high frequencies, the weighting function is very close to the *E*_ZPV_/*E*_th_ ratio. It follows that high-frequency vibrations in organic compounds will contribute significantly to broadenings. Indeed, as shown in Figure 2, the distributions of the internal coordinates at 300 K are narrower when classical thermal equilibrium is enforced than the more realistic distributions arising from quantization. We note that considering additional quantum effects such as vibronic progressions can further improve the bands. We can also notice in Figure 8 that the quantized spectrum is smoother, i.e., features fewer peaks, showing that quantization effectively masks the finer structure of the classical spectrum. To give further confidence to our strategy (i.e., the final quantized spectra is obtained using the kernel functions with bandwidths obtained from eq 16), we have calculated the electronic spectrum of **Acr** for the quantized trajectory using the kernel-width applied for the classical trajectory (0.022 eV) with the same number of snapshots and we compare it to the spectrum obtained with the optimal kernel width (0.045 eV). The plots in the inset of Figure 8 clearly show that broadening is not sensitive to this variation of the kernel width. Note, however, that the nonoptimal kernel width uncovers a few small peaks that are smoothed out by the optimal Gaussian kernels.

The comparison of the calculated and experimental spectra indicates that the quantum corrections improve significantly the agreement with the experiment. Both the line shapes and the intensity ratios of the peaks in the quantum-corrected spectrum are closer to those in the measured spectrum. This clearly indicates that including quantizations can significantly enhance the agreement with the experiments. The 20–30 nm discrepancies in the prediction of peak maxima, however, have not been improved. We attribute this to the limitation of the functional employed in TDDFT calculations. Two additional remarks are warranted, however, regarding the broadenings: one is that global hybrid functionals (such as B3LYP employed here) tend to yield spurious excitations,^59^ which may cause further broadening (the analogous ensemble spectra calculated by the CAM-B3LYP functional featuring range separation are given in the SI). The other is that the experimental band around 520 nm is vibronically broadened as demonstrated by the comparison with the experimental emission spectrum given in the SI of ref (28).

**Figure 9:**
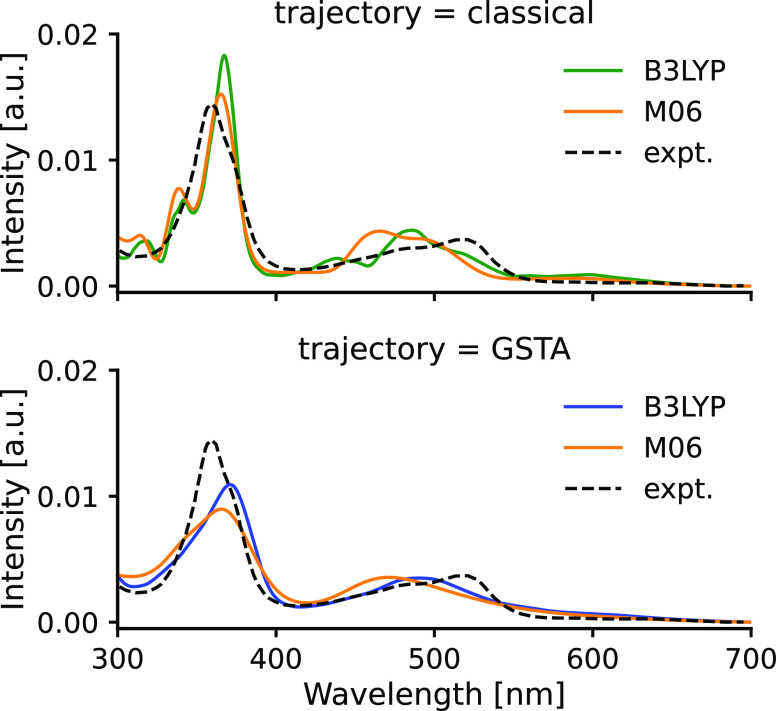
Comparison of the spectra calculated with B3LYP and M06 functionals for **Acr** in ACN solvent.

The functional tests showed that within the single-point approach, both functionals B3LYP and M06 performed equally well. In the following, we compare their performance for calculating the ensemble spectra. The spectra of the two classical ensembles shown in Figure 9 are quite similar, only a blueshift of 10–30 nm can be noticed in the M06 spectrum relative to B3LYP. The GSTA correction preserves this shift but the quantization broadens the bands by blurring together the smaller peaks for smoother spectra. Comparison of the two theoretical curves with the experiment shows that the B3LYP functional does a better job in the visible region, whereas the experimental peak around 360 nm is slightly better approximated by the M06 functional. Interestingly, for this single experimental peak, both functionals predict a composite band: an additional shoulder is computed for the quantized trajectories (at 330–340 nm), which, however, appears as a well-separated peak for the classical ensemble.

**Figure 10:**
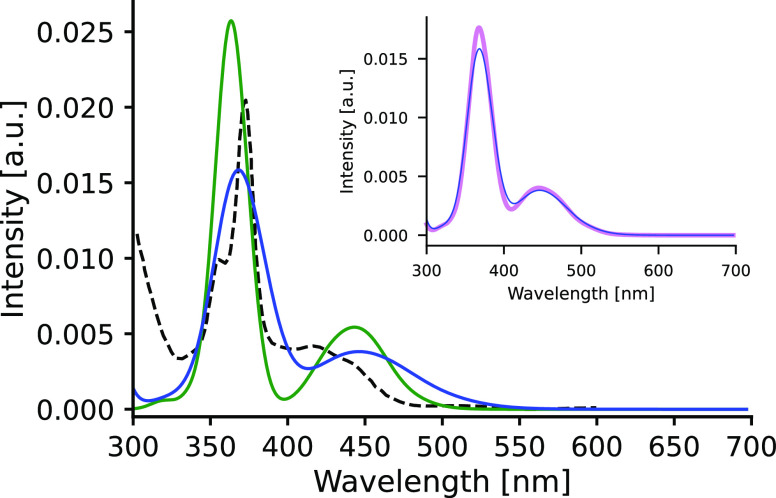
Comparison of theoretical spectra obtained from the classical (green) and from the GSTA-filtered (blue) trajectories of **Acs** in ACN solution. Inset: comparison of the GSTA-filtered spectra employing different kernel widths; violet: 0.061 eV; blue: 0.089 eV.

Figure 10 displays the spectra for the mesityl-acridinium salt **Acs** calculated for the classical and the GSTA trajectories with the B3LYP functional. We see again the broadening effect of the quantum corrections for both peaks around 450 and 360 nm and a hardly noticeable redshift of the peak around 360 nm. The inset convincingly shows that the variation of the kernel widths cannot be responsible for the pronounced broadening featured by the quantized spectra because a ca. 30% variation in the employed kernel width yields only marginal spectral changes.

As approximating the electronic spectra using only the excitation levels of a single stable configuration is a very popular approach to model the excited-state properties of molecules, we examine its performance in the present context. For the functional tests presented above, we have already followed this approach but here we give a few additional thoughts. In this approach, again, Gaussian functions are used to obtain smooth spectra from the stick bands of the computations; however, the width of these Gaussians accounting for the effects of environment and temperature is usually set empirically.^39^ Often, this broadening is estimated by neglecting the direct coupling between the solute and solvent nuclear degrees of freedom.^60,61^ Then, the final line shape can be obtained by convoluting the spectral line shapes of the solute (obtained, e.g., from the Franck–Condon approximation) with the solvent line shapes obtained from various approaches. For example, it is possible to treat the solvent as an ensemble of harmonic modes^13,60^ or within the framework of implicit solvent models to estimate the broadening due to the interaction between the solute and solvent.^61^ The latter approach yields a particularly simple formula based on Marcus theory:^63^ σ^2^ = 2*k*_B_*TE*_r_ = 2*k*_B_*T* (*E*_V_^neq^ – *E*_V_^eq^), where *E*_r_ is the reorganization energy of the solvent, and *E*_V_^neq^ and *E*_V_^eq^ are the contribution to the nonequilibrium and equilibrium free energies, respectively, at the given final electronic state. In the present case, this approximation with B3LYP yields σ = 0.03 eV for **Acr**, a much smaller value than the estimated broadening of 0.14 eV. This implies that the solvent-induced broadening represents a modest part of the overall broadening.

**Figure 11:**
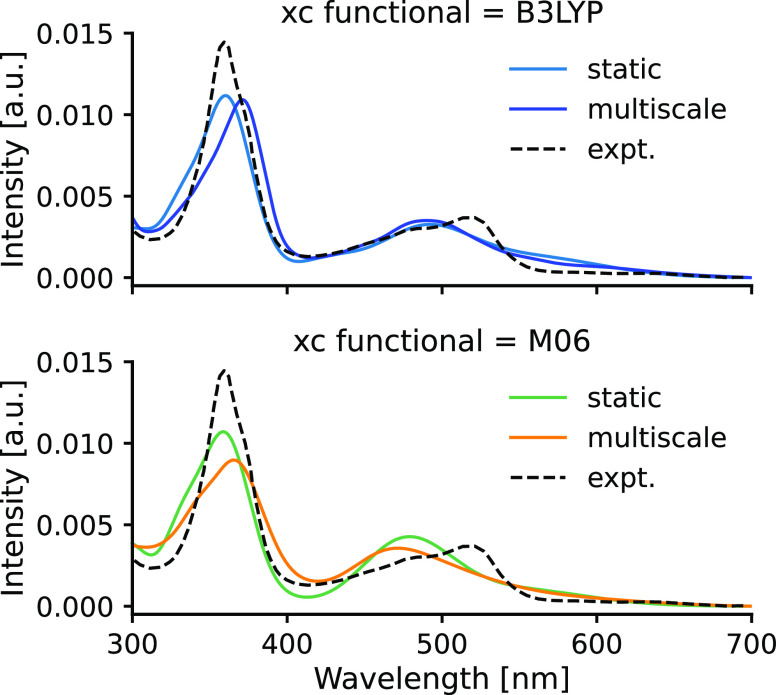
Comparison of the computed spectra obtained from the ensemble approach and for the optimized structure of **Acr** with functionals B3LYP (upper panel) and M06 (lower panel). The same comparison for **Acs** using the B3LYP functional is shown in Figure S4.

Using an optimal bandwidth of 0.14 eV, we can compare the results of the single-point approach with the quantum-corrected ensemble spectrum obtained from our multiscale approach. Figure 11 shows this comparison for both B3LYP and M06. We can see that the two methods yield very similar spectra for both functionals. In particular, the line shapes match quite well despite the huge difference in their Gaussian widths. A redshift is clearly seen for the peak at low wavelengths (360 vs 380 nm) of the ensemble spectra as compared to the spectrum obtained for the optimized configuration of **Acr**. Similar redshifts have been observed for other systems when nuclear quantum effects were included in the simulations.^12,14,15,64^ The good agreement between the two approaches warrants further discussion. First of all, these results can imply that enormous computational efforts could be saved by modeling the absorption spectra using the single-point approach within an error margin of a few tens of nanometers instead of employing our multiscale approach. However, we think that this good agreement between the two approaches is largely due to the noticeable core rigidity of the photocatalyst molecules (see Figure 3) and therefore this agreement is quite specific. This rigidity is an important ingredient for the delocalized electronic structure of these photocatalysts, which contributes mostly to the lowest transitions simulated here. We, therefore, argue that a similarly good agreement between single-point and ensemble approaches can be expected when the electronic spectrum corresponds to transitions of a conjugated system implying a certain structural rigidity. Examples where the ensemble approach significantly improves the spectrum obtained from the single-point approach can be found in the literature (see, e.g., refs (12) and (34)). Another aspect of this issue is that quantum effects are expected for high vibrational frequencies associated with strong bonds and rigid moieties in molecules. In contrast, flexibility gives rise to a large conformational variety with limited quantum effects but with a high degree of anharmonicity that prevents the harmonic Wigner sampling. As we have seen earlier in Figure 3, the quantum-corrected ensemble of **Acr** clearly indicates that our photocatalysts feature both rigid and flexible groups. Clearly, our selected molecules can serve as good model structures to exploit our multiscale approach in calculating quantum-corrected excitation spectra.

For the sake of completeness, we have compared the Franck–Condon and GSTA spectra for the first excitation and this comparison is given in the SI. The main conclusion is that the GSTA spectrum is redshifted and narrower than the FC spectrum. This difference is due to the fact that for significantly different configurations the nature of the first excitations might be different. In addition, the calculation of the FC spectrum requires additional approximations not used in the case of GSTA (zero temperature, harmonic PES for both the ground and the excited states, Condon approximation).

Finally, having an overall picture of the performance of the whole methodology, we wish to address the performance of GSTA to capture the vibrational broadenings. To this end, we have compared the GSTA spectrum against the Franck–Condon (FC) spectrum using a displaced harmonic oscillator (DHO) model, where a number of error sources (e.g., choice of the DFT functional, implicit solvent) are eliminated. This analysis is reported in the Supporting Information. For the DHO model the GSTA, the Wigner sampling and Truhlar’s LQ2 method gives identical Gaussian line shape for the absorption spectrum. They cannot reproduce the vibrational fine structure of the FC spectrum but they give the exact mean and standard deviation, which means that GSTA reproduces the exact broadening for the DHO model.

## IV Conclusions

In this work, we present a multiscale approach to obtain reliable absorption spectra that includes nuclear quantum effects for selected photocatalyst molecules. Our strategy is based on an extensive QM/MM sampling of solvated photocatalyst–solvent distributions. Subsequently, the trajectories are modified by the recently developed GSTA method to account for the nuclear quantum effects through a postprocessing treatment of the classical MD trajectories. The absorption spectrum is then obtained for both the classical and quantum trajectories by performing TDDFT calculations on the ensemble of solutes solvated implicitly in the same solvent. We also present a statistical method to find the optimal kernel function widths to obtain absorption spectra where the nuclear quantum effects can be identified and separated from the effects of artificial broadenings from kernel regression; this optimal width is a function of the size of the ensemble but it is optimal in the leave-one-out cross-validation sense.

A single-point (i.e., optimizing the molecular geometry and then performing a single TDDFT calculation) evaluation of typical functionals for TDDFT led to the conclusion that the B3LYP and the M06 functionals predict the absorption spectra of the selected photocatalysts sufficiently well. We also saw that without exact exchange or with additional Coulomb range-separation in the functionals, the agreement between theory and experiment is less satisfactory. Analysis of the spectra calculated with B3LYP and M06 in the ensemble average has clearly shown that quantum effects remarkably change the calculated absorption spectra: they broaden the absorption bands and conceal smaller peaks. We have also found that this feature is essential to achieve good agreement with the experiment. In this regard, we can conclude that the GSTA method represents a viable and easy-to-use method for revealing quantum effects influencing the electronic absorption spectra.

Interestingly, we have also found that for **Acr** and **Acs** the single-point approach performs equally well with an empirical broadening factor of 0.14 eV. This observation can be explained by noticing that the conjugated electronic structure of these photocatalysts induces a high degree of rigidity for these molecules, which restricts the configurational space of both molecules. Hence, similar good performance of the single-point approach cannot be expected for more flexible molecules. We also note that this single-point approach has limited predictive capability as the selection of the optimal broadening parameter has a significant influence on the absorption line shape and it cannot be determined adequately without experimental reference.

In conclusion, we have demonstrated that our multiscale approach can successfully predict optical spectra for photocatalyst molecules. Our approach features two new aspects as compared to other multiscale ensemble approaches: the way we obtain quantum-corrected nuclear coordinates and the new method to obtain the final spectrum from the individual peaks. The effect of quantum corrections is included in our methodology by employing the GSTA method on classical trajectories in a simple postprocessing method representing negligible computational cost. Further improvements can be anticipated by taking into account the dynamics of the ground and excited states via the dipole correlation functions. Our analysis indicated that GSTA can be applied successfully on anharmonic systems where the Wigner sampling does not work. It implies that the GSTA can also be used to generate initial conditions for photochemical dynamics calculations. We have also given a statistical protocol to obtain the width of the line shape function for producing ensemble-averaged spectra, discriminating in this way between the broadening due to quantization and the inevitable numerical effects.

## Supporting Information Available

Plots of the cost functions used to obtain the optimal bandwidths for the optical spectra of the ensembles (classical and quantized); spectra obtained through the single-point approach using bandwidths in the range of 0.04–0.15 eV; 12 spectra obtained from 400 randomly selected frames of the GSTA-corrected trajectory of **Acr**; comparison of the computed spectra of **Acs** obtained from the ensemble approach and from single-point approach using the B3LYP functional; spectra with molar extinction coefficients; comparison of the Franck–Condon spectrum with the absorption spectra of quantum and classical ensembles (PDF)
