# Supplementary material for: Multiscale Modeling of Electronic Spectra Including Nuclear Quantum Effects
Source: J Chem Theory Comput. 2021 Sep 28;17(10):6340–52. doi: 10.1021/acs.jctc.1c00531 (PMC8515811; doi:10.1021/acs.jctc.1c00531)
Supplement: Supplementary file 1 — ct1c00531_si_001.pdf [file ct1c00531_si_001.pdf]

Multiscale Modeling of Electronic Spectra Including Nuclear Quantum Effects

Péter P. Fehér*[1], Ádám Madarász*[1], and András Stirling*[1,2]

[1]Institute of Organic Chemistry, Research Centre for Natural Sciences, Magyar tudósok körútja. 2, Budapest, 1117, Hungary

[2]Department of Chemistry, Eszterházy Károly University, Leányka u. 6, 3300 Eger, Hungary

E-mail: feher.peter@ttk.hu, madarasz.adam@ttk.hu, stirling.andras@ttk.hu

List of contents

Fig. S1	Plot of the cost functions used to determine the optimal Gaussian bandwidth of each trajectory under study	Page S3
Fig. S2	Comparison of the spectra obtained from the single point convolution approach for **Acr** in ACN solution using Gaussian widths of 0.04-0.15 eV	Page S4
Fig. S3	Spectra obtained from 400 randomly selected frames from the GSTA corrected trajectory of **Acr**. The random selection has been carried out 12 times	Page S5
Fig. S4	Comparison of the computed spectra obtained from the ensemble approach and for the optimized structure of **Acs** with the B3LYP functional	Page S6
Fig. S5-10	Spectra with molar extinction coefficients	Page S10
Fig. S11-12	Comparison of Franck-Condon spectrum with the absorption spectra of quantum and classical ensembles	Page S11
	Coordinates of **Acr** and **Acs**	Page S18

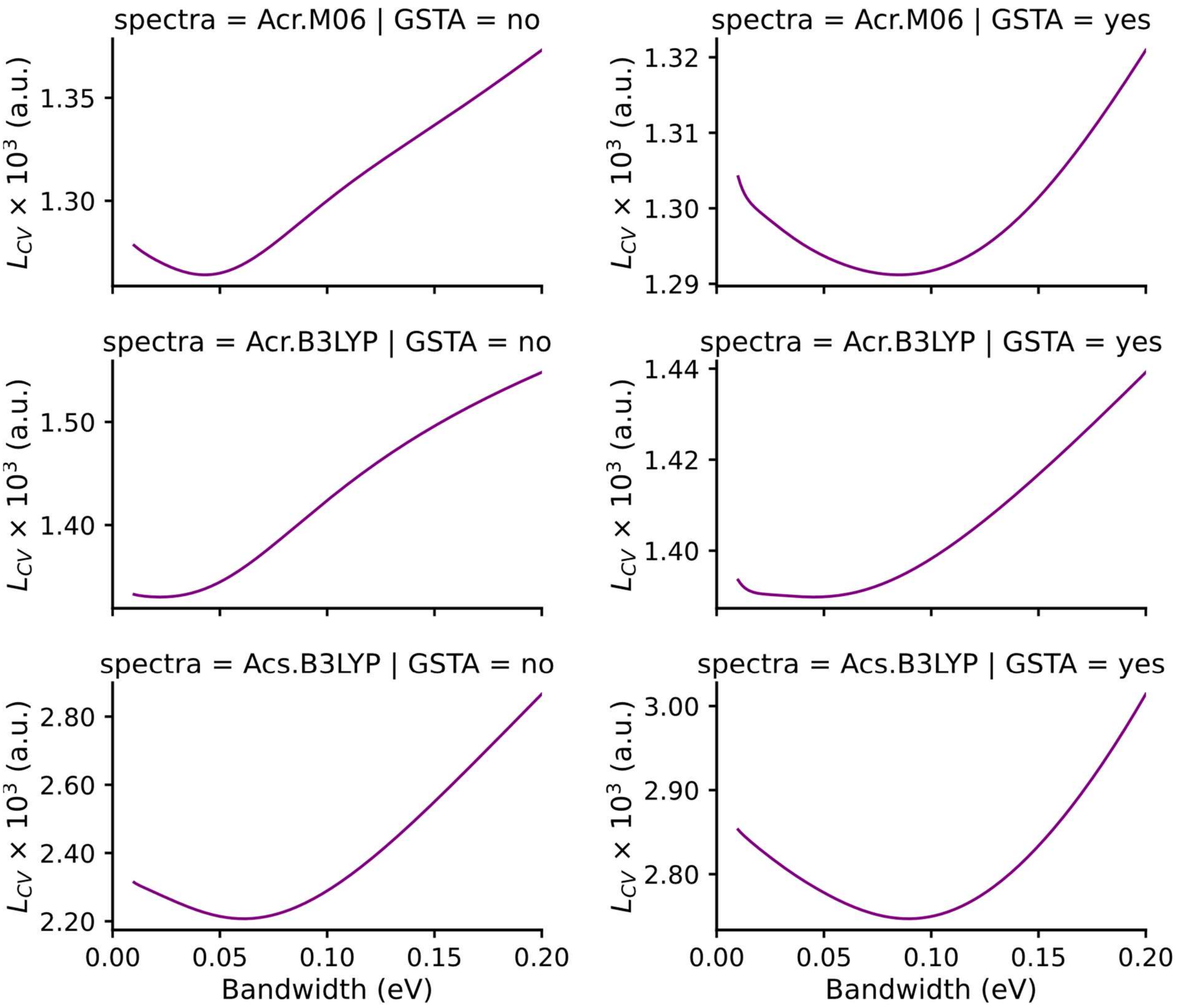

Figure S1. Plot of the cost function (eq. 16) used to determine the optimal Gaussian bandwidth (the minimum of each plot) of each trajectory under study.

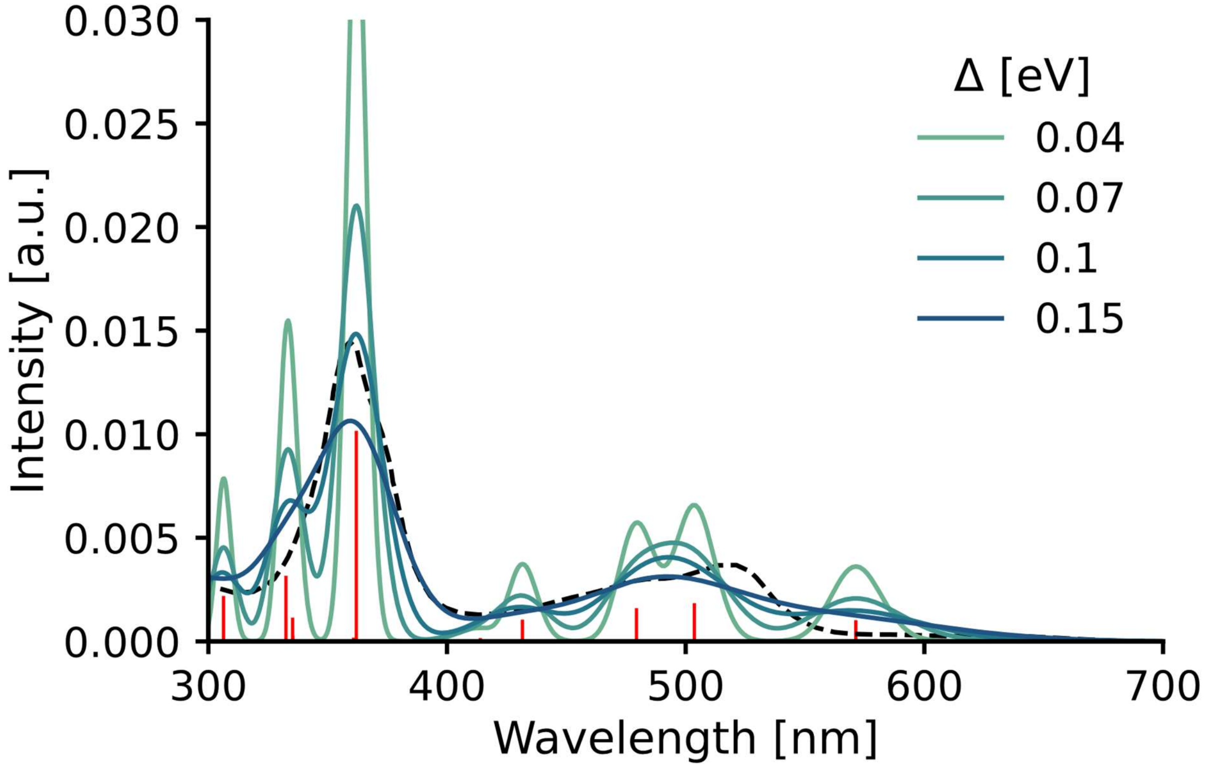

Figure S2. Comparison of the spectra obtained from the single point convolution approach for **Acr** in ACN solution using Gaussian widths of 0.04, 0.07, 0.1 and 0.15 eV. The stick spectrum in red represents the calculated excitations without Gaussian broadening. The dashed spectrum in black corresponds to the experimental spectrum.

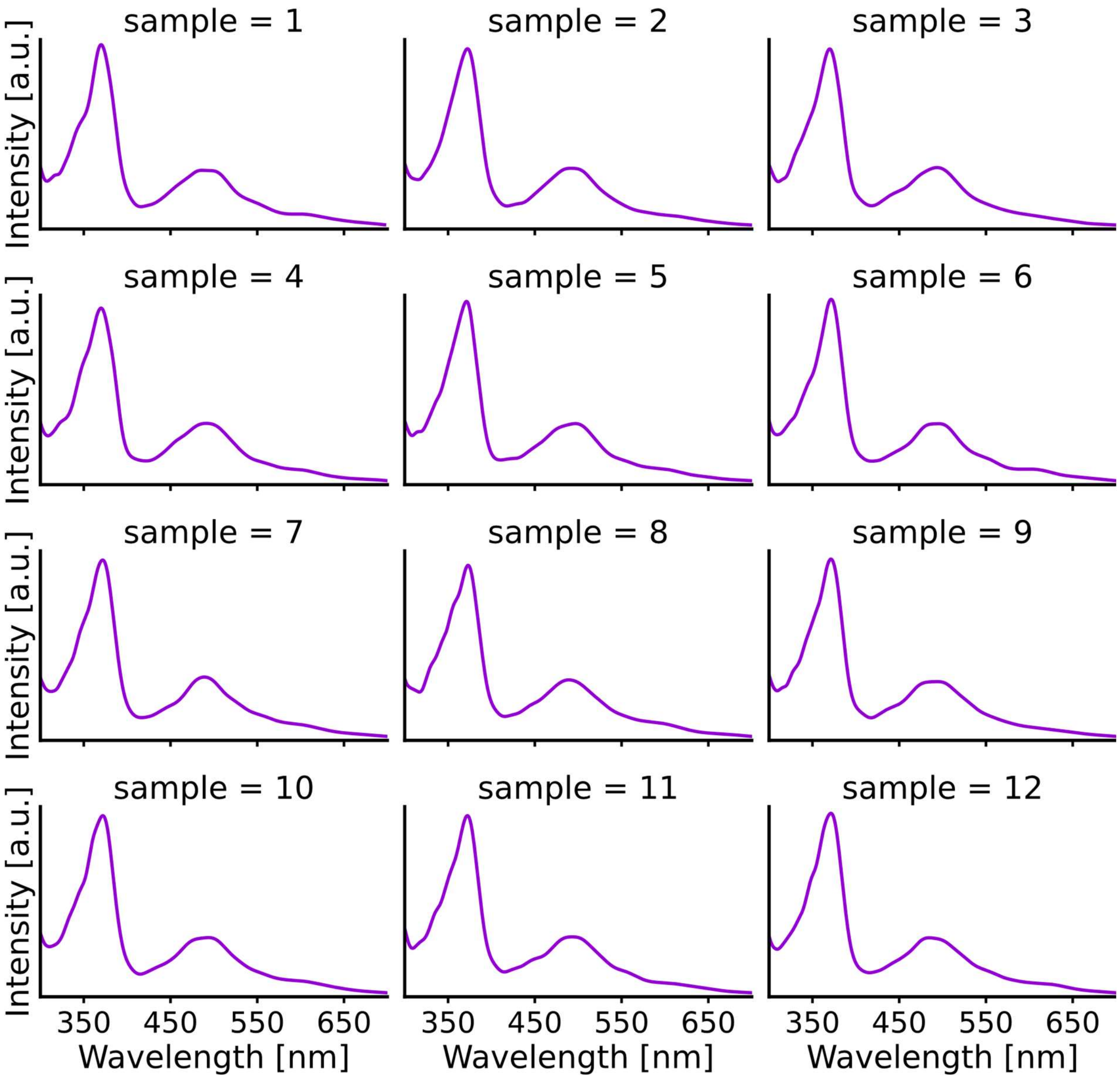

Figure S3. Spectra obtained from 400 randomly selected frames from the GSTA corrected trajectory of **Acr**. The random selection has been carried out 12 times.

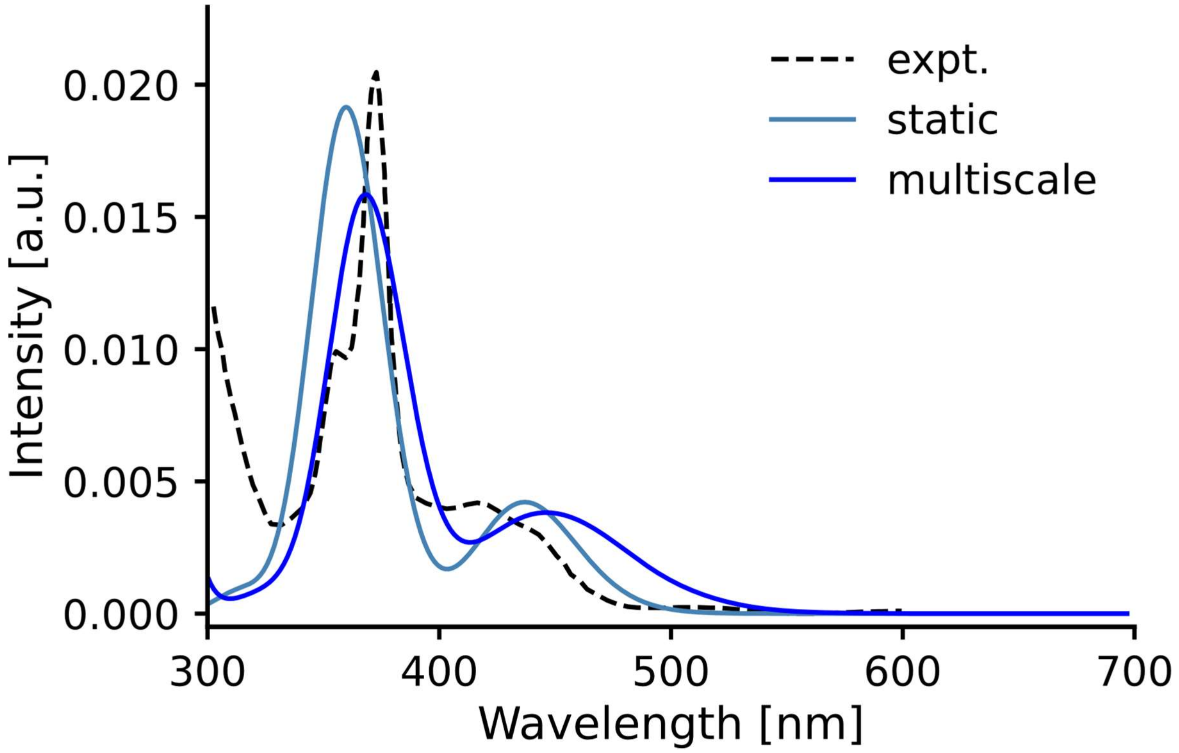

Figure S4. Comparison of the computed spectra obtained from the ensemble approach and for the optimized structure of **Acs** with the B3LYP functional.

Spectra with molar extinction coefficients

As our method is based on TDDFT, the obtained oscillator strengths (*f*) can be used to calculate the molar absorptivity (ε) as a function of the excitation energy or wavelength. We have used the following formula,

$$\varepsilon(E) = \frac{\pi}{2\ln(10)} \times \frac{N_A e^2 \hbar}{m_e c \epsilon_0} \times \sum_i \frac{f_i}{\Delta\sqrt{2\pi}} e^{-\frac{1}{2}\left(\frac{E-E_i}{\Delta}\right)^2}$$

where the second factor contains Avogadro's number (N_A), the elementary charge (*e*), the reduced Planck constant ($\hbar$), the electron mass (m_e), the speed of light (*c*) and the vacuum permittivity (ϵ_0). The third factor describes the sum of normalized (to unit area) Gaussian functions centered at the excitation energy of the individual excited states, multiplied by the corresponding oscillator strengths. The value of the expression before the sum is approximately 28706.7 M cm^{-1} eV.

To obtain the molar absorptivity as a function of wavelength, only the horizontal axis was transformed from eV to nm. Jacobi transformation was not used to preserve the area under the curve as absorption cross sections are typically determined by ratiometric measurements.

Note that the molar absorptivity depends significantly on the Δ parameter, therefore selecting its optimal value is crucial to obtain proper agreement with experiment. Unfortunately, Ref. 28 only provides normalized spectra or spectra without concentrations, therefore we were unable to assess the accuracy of the calculated values.

We also include here the **Acr** spectra calculated with CAM-B3LYP for comparison. We obtained rather high Δ values after optimization (0.136 and 0.227 for the classical and GSTA-corrected ensembles, respectively), which is likely due to the bad accuracy of the functional (Fig. 5). We tried redshifting the spectra by 50 nm, but it did not improve the results.

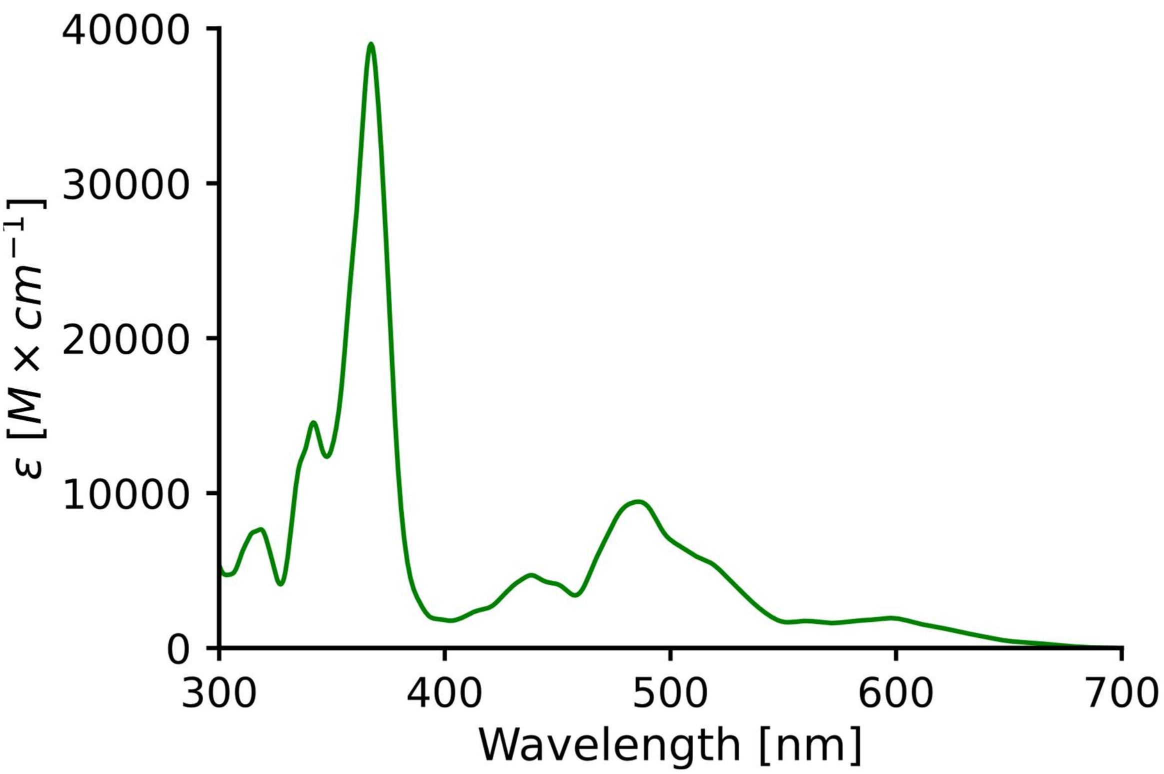

Figure S5. The classical ensemble averaged spectrum of **Acr** without normalization.

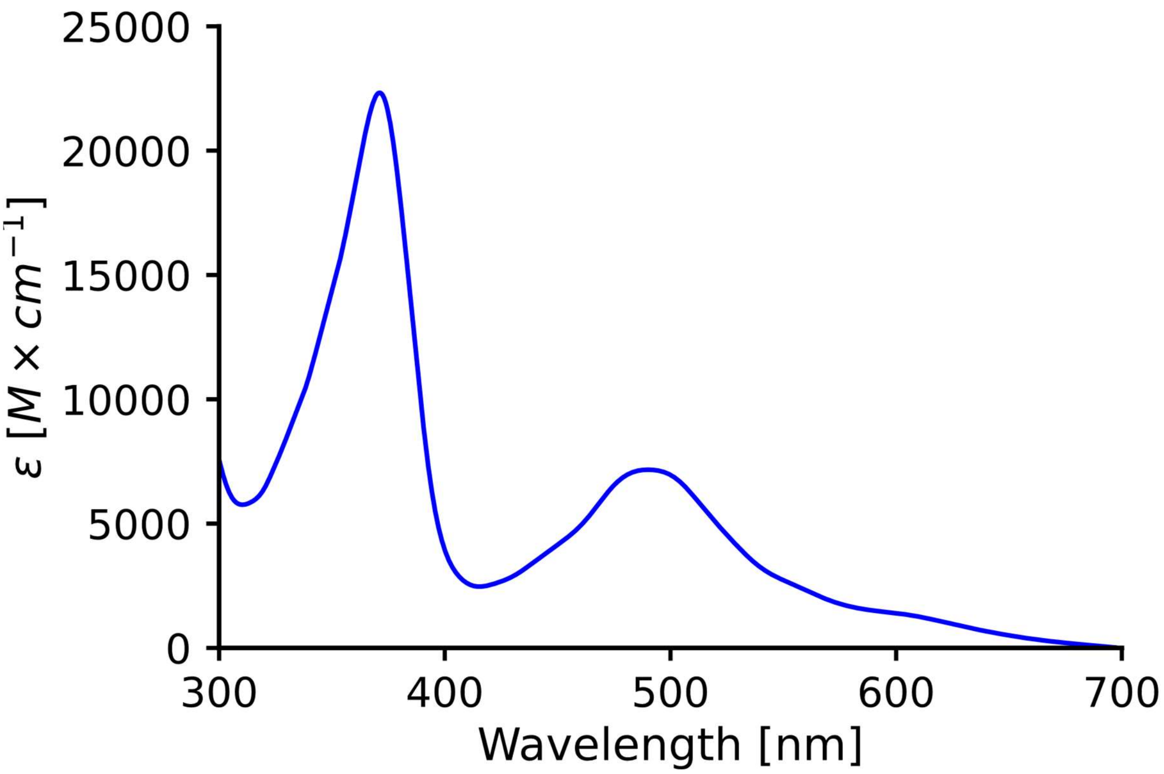

Figure S6. The GSTA ensemble averaged spectrum of **Acr** without normalization.

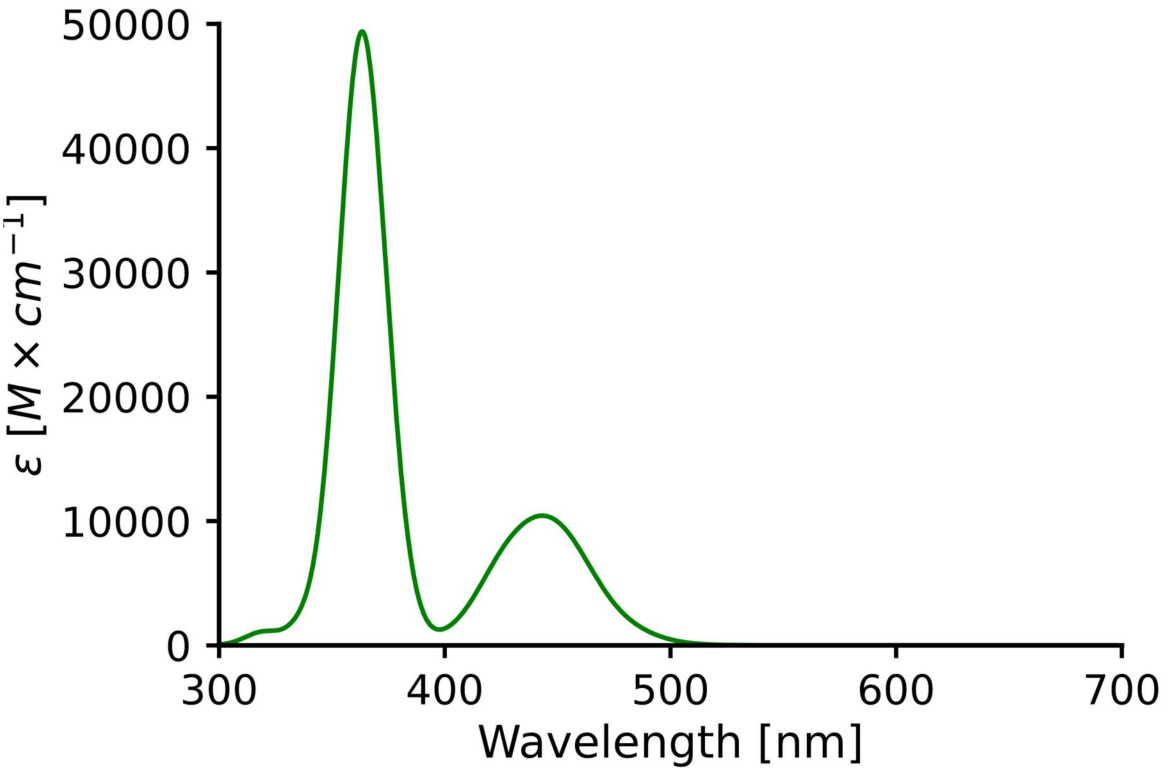

Figure S7. The classical ensemble averaged spectrum of **Acs** without normalization.

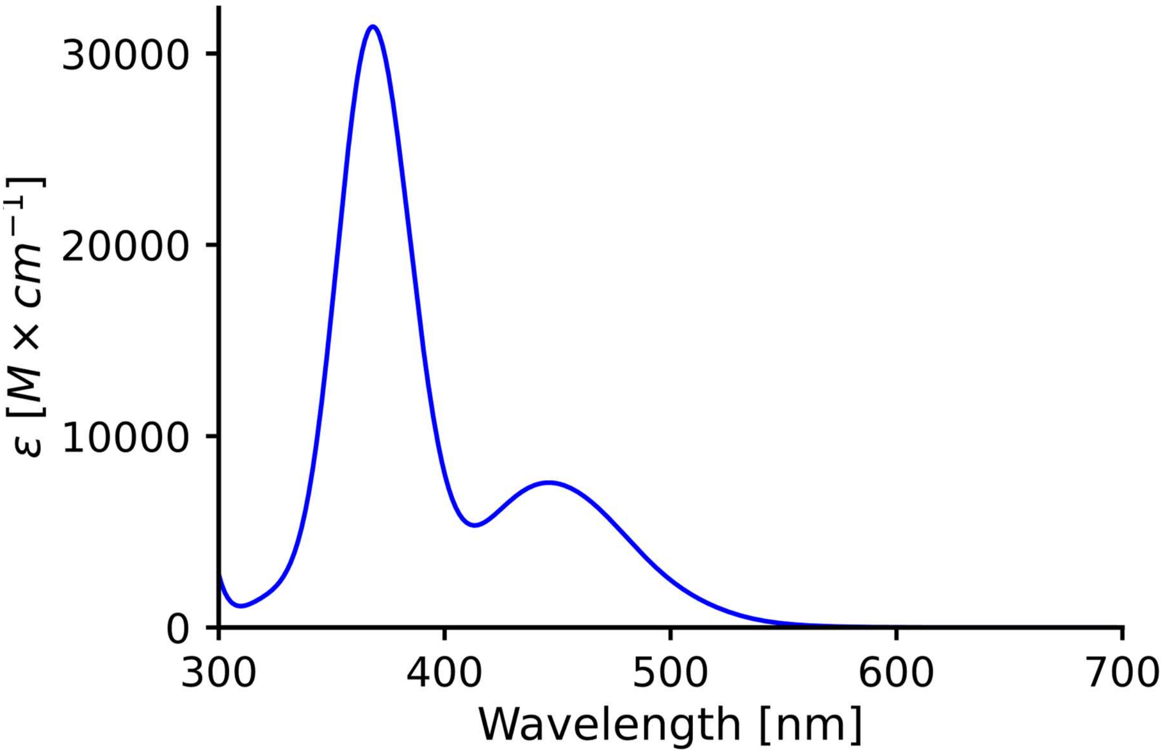

Figure S8. The GSTA ensemble averaged spectrum of **Acs** without normalization.

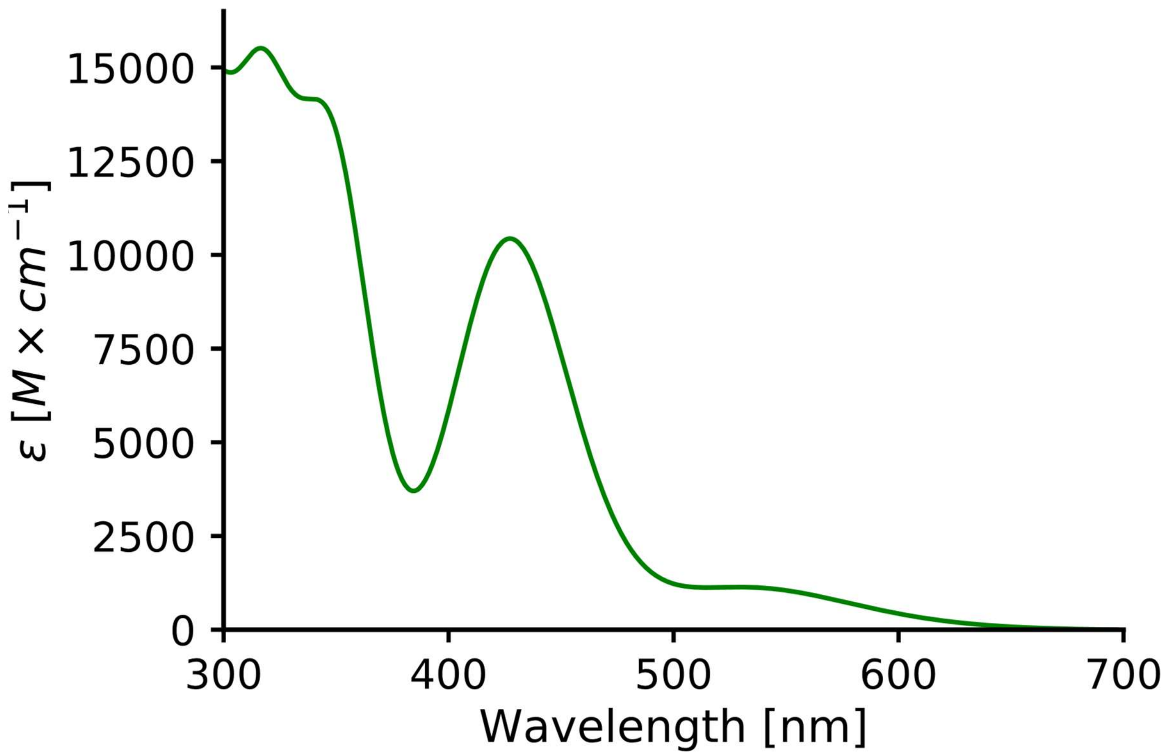

Figure S9. The classical ensemble averaged spectrum of **Acr** without normalization calculated with the CAM-B3LYP functional.

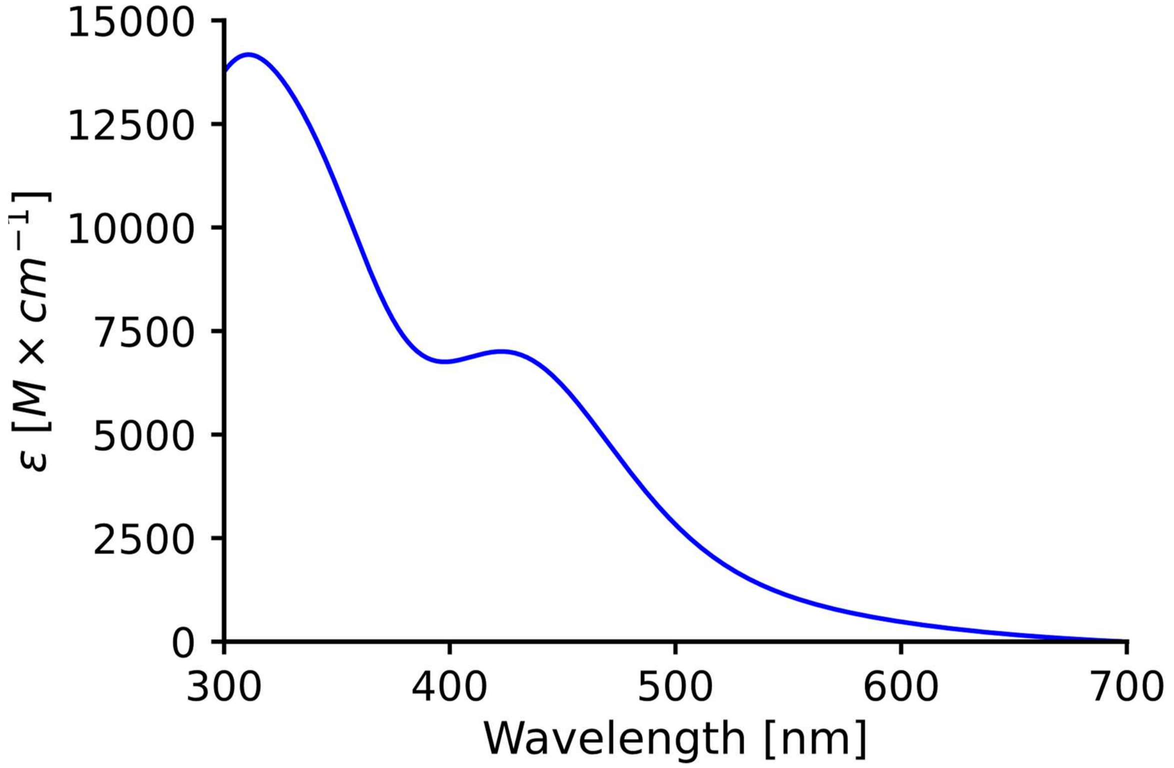

Figure S10. The GSTA ensemble averaged spectrum of **Acr** without normalization calculated with the CAM-B3LYP functional.

Comparison of Franck-Condon spectrum with the absorption spectra of quantum and classical ensembles

1. The Displaced Harmonic Oscillator (DHO) model

The Franck-Condon spectrum

For the comparison of different methods, we use the Displaced Harmonic Oscillator (DHO) model at zero temperature. The notations and the derivations are taken from Ref. [1].

The ground state Hamiltonian is

$$H_g = \frac{p^2}{2m} + \frac{1}{2} m\omega_0^2 q^2 \tag{1}$$

where q is the position, p is the momentum, m is the mass and ω_0 is the frequency. The excited state Hamiltonian H_e has the same form, but the ground state potential is displaced by d, and it is higher in energy by $\hbar\omega_{eg}$:

$$H_e = \frac{p^2}{2m} + \frac{1}{2} m\omega_0^2 (q-d)^2 + \hbar\omega_{eg} \tag{2}$$

$\hbar$ is the reduced Planck constant, $\hbar\omega_{eg} = E_e - E_g$, where E_e and E_g are the minimum energies of the excited and ground states, respectively.

Let us introduce the Huang-Rhys parameter:

$$D = \frac{d^2 m\omega_0}{2\hbar} \tag{3}$$

It has been shown that the Franck-Condon spectrum is:

$$\sigma_{abs}(\omega) = \left|\mu_{eg}\right|^2 \sum_{j=0}^{\infty} e^{-D} \frac{1}{j!} D^j \delta\left(\omega - \omega_{eg} - j\omega_0\right) \tag{4}$$

The intensities show a Poisson distribution.

It can be derived that the envelope of the spectrum is a Gaussian function:

$$\sigma_{abs}(\omega) = \frac{1}{\sqrt{2\pi D}\omega_0} \left|\mu_{eg}\right|^2 exp\left(-\frac{\left(\omega - \omega_{eg} - D\omega_0\right)^2}{2D\omega_0^2}\right) \tag{5}$$

Note that eq 5 is normalized to $\left|\mu_{eg}\right|^2$.

This Gaussian function was derived by Barbatti[2] and Truhlar[3] as well.

The Quantum ensemble

At zero temperature the probability density function of the position is:

$$\rho(q) = \sqrt{\frac{m\omega_0}{\pi\hbar}} exp\left(-\frac{\frac{1}{2} m\omega_0^2 q^2}{\frac{1}{2}\hbar\omega_0} \right) = \sqrt{\frac{m\omega_0}{\pi\hbar}} exp\left(-\frac{m\omega_0}{\hbar} q^2 \right) \tag{6}$$

If the position is q, then the energy gap corresponds to a photon with a frequency of

$$\omega = \frac{H_e - H_g}{\hbar} = \frac{m\omega_0^2 d^2}{2\hbar} - \frac{m\omega_0^2 dq}{\hbar} + \omega_{eg} = D\omega_0 - 2D\omega_0 \frac{q}{d} + \omega_{eg} \tag{7}$$

From this equation the position can be expressed:

$$q = \frac{D\omega_0 + \omega_{eg} - \omega}{2D\omega_0} d \tag{8}$$

For the absorption spectrum we need the probability density function of the frequency that can be obtained from eq 6 through Jacobian transformation:

$$\rho(\omega) = \left| \frac{\partial q}{\partial \omega} \right| \rho(q) = \frac{d}{2D\omega_0} \rho(q) = \frac{d}{2D\omega_0} \sqrt{\frac{m\omega_0}{\pi\hbar}} exp\left(-\frac{m\omega_0}{\hbar} q^2 \right) \tag{9}$$

Replacing q with eq 8:

$$\rho(\omega) = \frac{d}{2D\omega_0} \sqrt{\frac{m\omega_0}{\pi\hbar}} exp\left(-\frac{\left(\omega - \omega_{eg} - D\omega_0\right)^2}{2D\omega_0^2} \right) \tag{10}$$

Using eq 3 in the prefactor we obtain:

$$\rho(\omega) = \frac{1}{\sqrt{2\pi D}\omega_0} exp\left(-\frac{\left(\omega - \omega_{eg} - D\omega_0\right)^2}{2D\omega_0^2} \right) \tag{11}$$

If this function is multiplied with $\left|\mu_{eg}\right|^2$, we obtain the exact envelope of the Franck-Condon spectrum in eq 5.

It follows from the basic properties of the Poisson and Gaussian distributions that they have the same mean: $\omega_{eg} + D\omega_0$ and variance: $D\omega_0^2$.

The Classical ensemble

At zero temperature the absorption spectrum determined from classical model leads to a Dirac delta function at the frequency of $\omega_{eg} + D\omega_0$.

Conclusions

The absorption spectrum computed with GSTA does not reproduce the fine structure of the Franck-Condon spectrum, but the GSTA reproduces the exact mean and broadening within the Displaced Harmonic Oscillator (DHO) model. The classical ensemble reproduces the exact mean of the FC spectrum but not the broadening. The comparison of the different spectra is shown in Figure S11.

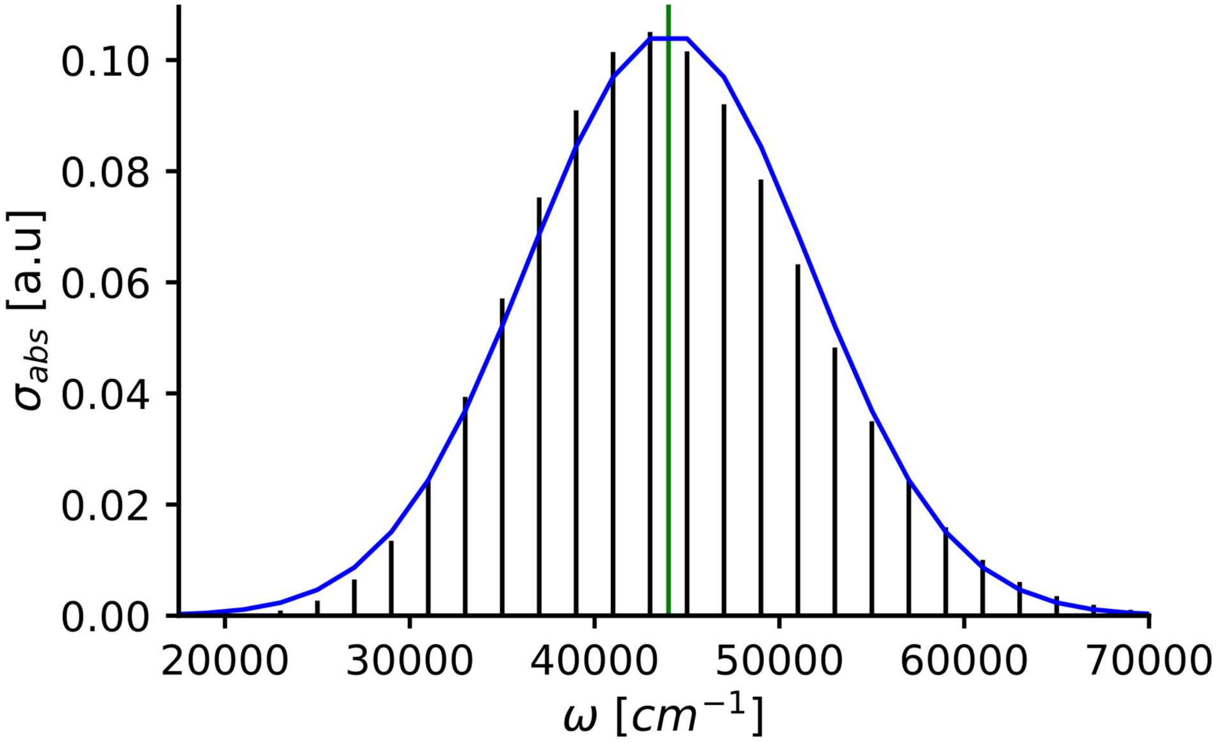

Figure S11. Simulated absorption spectrum of a Displaced Harmonic Oscillator (DHO) model. Parameters: ω_{eg}=15000 cm^{-1}, ω_0 =2000 cm^{-1}, D = 14.5. Black, blue and green lines correspond to the FC, quantum (GSTA) and classical approaches, respectively.

2. <u>*Comparison of the FC and GSTA spectrum for **Acr***</u>

To compare the FC and GSTA spectra, we investigated the first excitation of **Acr**. The spectra are shown in Fig. S12, while the mean values along with the standard deviations are collected in Table S1. The GSTA spectrum is red shifted and narrower than the FC spectrum. This difference is due to the fact that for significantly different configurations the nature of the first excitations can be different. Also, in the calculation of the FC spectrum requires additional approximations not used in the case of GSTA:

- zero temperature
- the PES is harmonic for both the ground state and the excited states
- transient dipole moment is independent from the nuclear coordinates (Condon approximation)

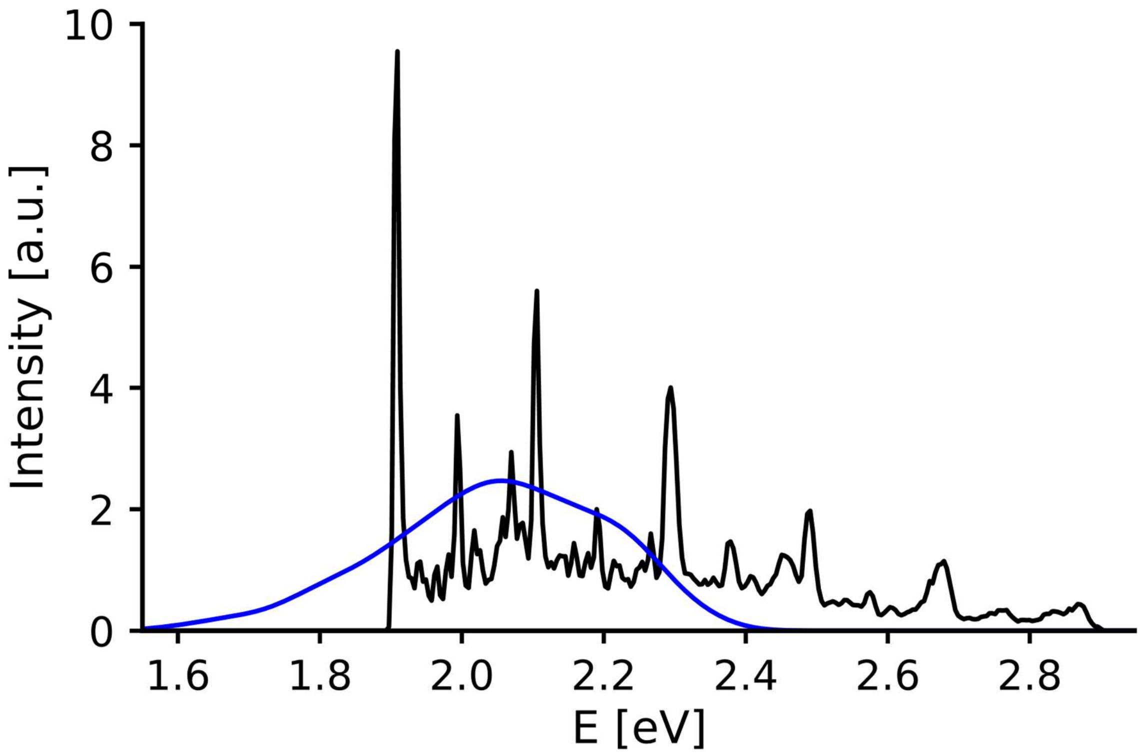

Figure S12. The absorption spectra simulated for the first excitation of **Acr**. The Δ values are 0.003 and 0.045 eV for FC (black line) and GSTA (blue line), respectively.

Table S1. Main properties of the simulated spectra for the first excitation.

	mean / eV	standard deviation / eV
FC spectrum	2.24	0.25
GSTA	2.05	0.15
Optimized geometry with B3LYP functional	2.20	0.0

To calculate the FC spectrum, we followed the tutorial of Barone.[4] The input files and the optimized geometries can be found below.

ground state optimization:

```
%chk=Mes-Acr_0d_B3LYP_opt.chk
#p opt(readfc) freq=noraman ub3lyp scrf=(smd,solvent=acetonitrile)
empiricaldispersion=gd3bj int=ultrafine tzvp geom=allcheck guess=tcheck
```

the final coordinates (in Angstroms, xyz format) of the optimized ground state:

```
77
./Mes-Acr_0d_B3LYP_opt_folyt.out Energy: -1451.386699 Hartree
C         -0.68470        3.66991       -0.00495
C         -1.33922        2.43287       -0.00258
C         -0.64277        1.22683       -0.00469
C          0.78280        1.22296       -0.00784
C          1.42483        2.47828       -0.01045
C          0.71445        3.66774       -0.00959
```

C	1.49318	-0.00722	-0.00774
C	0.77084	-1.23043	-0.00803
C	-0.65470	-1.22044	-0.00489
C	-1.36285	-2.41965	-0.00298
H	-2.44107	-2.38377	0.00124
C	-0.72038	-3.66297	-0.00542
C	0.67872	-3.67443	-0.01010
C	1.40062	-2.49194	-0.01087
H	-2.41774	2.40734	0.00173
H	2.50653	2.50069	-0.01341
H	1.26372	4.59819	-0.01204
H	1.21894	-4.61016	-0.01265
H	2.48204	-2.52498	-0.01386
N	-1.32195	0.00648	-0.00296
C	-2.75885	0.01355	-0.00204
C	-3.44489	0.01694	1.20663
C	-3.44589	0.01723	-1.21016
C	-4.83591	0.02415	1.20516
H	-2.88784	0.01402	2.13494
C	-4.83687	0.02445	-1.20764
H	-2.88955	0.01453	-2.13890
C	-5.53193	0.02788	-0.00096
H	-5.37506	0.02681	2.14422
H	-5.37674	0.02734	-2.14629
H	-6.61490	0.03348	-0.00054
C	2.97984	-0.01449	-0.00341
C	3.67826	-0.02181	1.21478
C	3.68729	-0.02015	-1.21589
C	5.07285	-0.03300	1.20115
C	5.08212	-0.03129	-1.19159
C	5.79352	-0.03505	0.00738
H	5.60773	-0.04201	2.14503
H	5.62419	-0.03898	-2.13133
C	-1.51972	4.95329	0.00402
C	-1.56800	-4.93805	0.00356
C	-0.70745	-6.20695	-0.02825
H	-0.08895	-6.25394	-0.92713
H	-0.05159	-6.27373	0.84230
H	-1.35659	-7.08508	-0.02389
C	-2.42959	-4.96836	1.28107
H	-1.79993	-4.96178	2.17407
H	-3.09990	-4.10880	1.33295
H	-3.04082	-5.87436	1.30160
C	-2.49355	-4.95007	-1.22853
H	-3.17209	-4.09558	-1.23103
H	-1.91062	-4.92315	-2.15234
H	-3.09926	-5.85989	-1.23434
C	-0.64659	6.21356	-0.02793
H	0.00985	6.27391	0.84265
H	-0.02762	6.25427	-0.92679
H	-1.28696	7.09812	-0.02371
C	-2.44517	4.97452	-1.22802
H	-1.86257	4.94219	-2.15186
H	-3.13195	4.12665	-1.23075
H	-3.04209	5.89015	-1.23360
C	-2.38087	4.99233	1.28160
H	-3.05943	4.13929	1.33380
H	-1.75122	4.97992	2.17454
H	-2.98332	5.90419	1.30192
C	2.93286	-0.02184	2.52262
H	2.29105	0.85809	2.61273
H	2.28557	-0.89809	2.60988
H	3.62558	-0.02536	3.36470
C	2.95193	-0.01841	-2.52936
H	2.30451	-0.89399	-2.62227
H	2.31166	0.86223	-2.62360
H	3.65096	-0.02185	-3.36620
C	7.29893	-0.01150	0.01416

```
H          7.67249        1.01685        0.04977
H          7.70039       -0.53304        0.88494
H          7.70743       -0.47568       -0.88512
```

excited state optimization:

```
%chk=Mes-Acr_0d_B3LYP_opt_S1.chk
#p TD(nstates=25,root=1) opt(readfc) freq=(noraman,SaveNM) ub3lyp
scrf=(smd,solvent=acetonitrile) empiricaldispersion=gd3bj int=ultrafine tzvp
geom=allcheck guess=tcheck
```

final coordinates of the first excited state:

```
77
./Mes-Acr_0d_B3LYP_opt_S1.out    Energy: -1451.314537 Hatree
C         -0.71320       -3.64667       -0.02450
C         -1.36064       -2.43181       -0.00882
C         -0.66279       -1.20750       -0.00120
C          0.79827       -1.21257       -0.01031
C          1.43912       -2.48485       -0.03592
C          0.73895       -3.65277       -0.04051
C          1.48752        0.00440        0.00918
C          0.79053        1.21693        0.03117
C         -0.67044        1.20274        0.02002
C         -1.37593        2.42266        0.02636
H         -2.45471        2.39044        0.01132
C         -0.73610        3.64148        0.04756
C          0.71596        3.65667        0.06337
C          1.42343        2.49319        0.05702
H         -2.43967       -2.40607       -0.00631
H          2.52138       -2.50629       -0.04897
H          1.27415       -4.58982       -0.05727
H          1.24526        4.59699        0.08385
H          2.50553        2.52142        0.07051
N         -1.31737       -0.00443        0.00903
C         -2.76283       -0.00905        0.00907
C         -3.43669       -0.00651       -1.20275
C         -3.43613       -0.01610        1.22113
C         -4.83189       -0.01092       -1.19898
H         -2.87557       -0.00120       -2.12819
C         -4.83127       -0.02106        1.21815
H         -2.87453       -0.01774        2.14630
C         -5.52378       -0.01830        0.00972
H         -5.37271       -0.00871       -2.13677
H         -5.37155       -0.02688        2.15623
H         -6.60695       -0.02193        0.00998
C          2.97713        0.00937        0.00489
C          3.66906        0.16884       -1.20508
C          3.67828       -0.13920        1.21084
C          5.06383        0.17144       -1.18837
C          5.07291       -0.12384        1.18566
C          5.78406        0.02565       -0.00385
H          5.59796        0.29278       -2.12464
H          5.61417       -0.22996        2.11968
C         -1.53278       -4.93978       -0.05106
C         -1.56349        4.92977        0.03160
C         -0.68838        6.18566        0.14002
H         -0.10636        6.19477        1.06411
H          0.00268        6.27337       -0.70065
H         -1.32640        7.07199        0.13888
C         -2.35813        5.00619       -1.28804
H         -1.68376        5.00417       -2.14779
H         -3.03998        4.16008       -1.38975
H         -2.95020        5.92497       -1.32136
C         -2.55295        4.93156        1.21363
```

```
H          -3.24676        4.09124        1.16036
H          -2.02010        4.87189        2.16568
H          -3.14151        5.85290        1.20982
C          -0.64816       -6.19162        0.02145
H           0.02867       -6.26119       -0.83236
H          -0.05008       -6.21191        0.93508
H          -1.28059       -7.08193        0.01674
C          -2.50386       -4.96960        1.14574
H          -1.95687       -4.92322        2.09048
H          -3.20432       -4.13358        1.11827
H          -3.08608       -5.89490        1.13430
C          -2.34715       -4.99756       -1.35956
H          -3.03558       -4.15405       -1.43542
H          -1.68610       -4.97576       -2.22932
H          -2.93409       -5.91935       -1.40049
C           2.93122        0.35120       -2.50494
H           2.13929       -0.39087       -2.62417
H           2.45852        1.33591       -2.55500
H           3.61367        0.26387       -3.35064
C           2.95031       -0.32127        2.51625
H           2.15602        0.41768        2.63936
H           2.48221       -1.30785        2.57185
H           3.63829       -0.22913        3.35694
C           7.28913        0.00031       -0.01304
H           7.66021       -1.02094       -0.14560
H           7.69122        0.60154       -0.83030
H           7.69799        0.37595        0.92653
```

calculation of the FC spectrum:

```
%chk=Mes-Acr_0d_B3LYP_opt.chk
#p geom=AllCheck Freq=(ReadFC,FC,SaveNM,ReadFCHT) NoSymm

 PRTINT=0.00000000000000000001 PRTMAT=12 MAXC1=100

Mes-Acr_0d_B3LYP_opt_S1.chk
```

References:

[1] https://ocw.mit.edu/courses/chemistry/5-74-introductory-quantum-mechanics-ii-spring-2009/lecture-notes/MIT5_74s09_lec08.pdf

[2] Crespo-Otero, R.; Barbatti, M. Spectrum Simulation and Decomposition with Nuclear Ensemble: Formal Derivation and Application to Benzene, Furan and 2-Phenylfuran. *Theor. Chem. Acc.* **2012**, *131*, 1237.

[3] Li, S. L.; Truhlar, D. G. Franck–Condon Models for Simulating the Band Shape of Electronic Absorption Spectra. *J. Chem. Theory Comput.* **2017**, *13*, 2823–2830.

[4] https://smart.sns.it/pdf/vibronic_spectra_G09-A02.pdf

Coordinates (in Å) of **Acr** and **Acs**

```
77
Acr, PBE-D3 geometry
C          0.69223        3.68373       -0.00497
C          1.34869        2.43991       -0.00243
C          0.64522        1.22980       -0.00432
C         -0.78786        1.22678       -0.00726
C         -1.43070        2.48954       -0.01013
C         -0.71416        3.68323       -0.00958
C         -1.50080       -0.00665       -0.00702
C         -0.77674       -1.23362       -0.00742
C          0.65630       -1.22373       -0.00449
C          1.37064       -2.42748       -0.00278
H          2.45923       -2.38848        0.00131
C          0.72539       -3.67711       -0.00529
C         -0.68095       -3.68931       -0.00986
C         -1.40818       -2.50210       -0.01035
H          2.43759        2.41057        0.00171
H         -2.52210        2.51085       -0.01308
H         -1.26418        4.62441       -0.01234
H         -1.22250       -4.63537       -0.01262
H         -2.49935       -2.53330       -0.01326
N          1.32353        0.00607       -0.00264
C          2.76462        0.01267       -0.00176
C          3.45408        0.01614       -1.21722
C          3.45319        0.01580        1.21419
C          4.85159        0.02287       -1.21340
H          2.89262        0.01364       -2.15337
C          4.85074        0.02252        1.21127
H          2.89112        0.01303        2.14996
C          5.54958        0.02603       -0.00082
H          5.39595        0.02560       -2.15967
H          5.39448        0.02498        2.15789
H          6.64137        0.03125       -0.00044
C         -2.98918       -0.01336       -0.00284
C         -3.70057       -0.02022       -1.22295
C         -3.69173       -0.01963        1.22238
C         -5.10207       -0.03096       -1.19722
C         -5.09342       -0.03043        1.20677
C         -5.81848       -0.03302        0.00742
H         -5.64792       -0.04002       -2.14556
H         -5.63244       -0.03914        2.15899
C          1.52954        4.96797        0.00323
C          1.57434       -4.95368        0.00343
C          0.71245       -6.22404       -0.02756
H          0.05192       -6.29183        0.85012
H          0.08867       -6.27251       -0.93308
H          1.36680       -7.10878       -0.02316
C          2.50137       -4.96399       -1.23069
H          1.91513       -4.94203       -2.16231
H          3.18152       -4.09963       -1.23472
H          3.11604       -5.87793       -1.23353
C          2.43878       -4.98161        1.28214
H          3.11084       -4.11238        1.33421
H          1.80653       -4.97919        2.18345
H          3.05883       -5.89182        1.30008
C          0.65598        6.23039       -0.02650
H          0.03086        6.27339       -0.93136
H         -0.00421        6.29178        0.85189
H          1.30217        7.12110       -0.02251
C          2.39514        5.00374        1.28096
H          1.76394        4.99569        2.18297
H          3.07507        4.14063        1.33239
H          3.00698        5.91948        1.29817
```

C	2.45499	4.98706	-1.23198
H	3.14252	4.12857	-1.23739
H	1.86783	4.96061	-2.16291
H	3.06177	5.90625	-1.23503
C	-2.96437	-0.02069	-2.53680
H	-2.31626	0.86477	-2.63319
H	-2.31147	-0.90300	-2.63023
H	-3.66745	-0.02400	-3.38102
C	-2.94635	-0.01932	2.53109
H	-2.29184	-0.90089	2.62004
H	-2.29854	0.86684	2.62299
H	-3.64361	-0.02333	3.38012
C	-7.32466	-0.00816	0.01286
H	-7.70107	1.02853	0.00278
H	-7.73814	-0.51229	-0.87262
H	-7.73118	-0.49291	0.91221

77

Acs, PBE-D3 geometry

C	0.69385	3.64935	-0.01212
C	1.35909	2.42924	-0.00614
C	0.65325	1.21101	-0.00952
C	-0.78223	1.21275	-0.01509
C	-1.44159	2.47322	-0.02113
C	-0.73001	3.64954	-0.02181
C	-1.49067	-0.00732	-0.01378
C	-0.77049	-1.22053	-0.01495
C	0.66496	-1.20497	-0.00966
C	1.38248	-2.41636	-0.00683
H	2.47010	-2.38065	0.00198
C	0.72891	-3.64277	-0.01261
C	-0.69488	-3.65668	-0.02171
C	-1.41774	-2.48729	-0.02086
H	2.44697	2.40372	0.00327
H	-2.53205	2.48435	-0.02656
H	-1.27112	4.59443	-0.02893
H	-1.22686	-4.60673	-0.02871
H	-2.50806	-2.50903	-0.02611
N	1.32532	0.00625	-0.00602
C	2.77832	0.01345	-0.00254
C	3.45776	0.01790	-1.22091
C	3.45349	0.01632	1.21816
C	4.85480	0.02550	-1.21212
H	2.89587	0.01554	-2.15631
C	4.85061	0.02391	1.21404
H	2.88847	0.01282	2.15168
C	5.54927	0.02849	0.00216
H	5.40024	0.02903	-2.15730
H	5.39288	0.02627	2.16104
H	6.64083	0.03442	0.00403
C	-2.97869	-0.01428	-0.00550
C	-3.68310	-0.01941	-1.22807
C	-3.66464	-0.02201	1.22771
C	-5.08326	-0.03020	-1.19229
C	-5.06515	-0.03279	1.21292
C	-5.79344	-0.03394	0.01578
H	-5.63196	-0.03799	-2.13839
H	-5.59957	-0.04266	2.16712
C	1.50900	4.94532	0.00507
C	1.55661	-4.93079	0.00409
C	0.68047	-6.18929	-0.07078
H	-0.00064	-6.26757	0.78971
H	0.08091	-6.21639	-0.99317
H	1.32831	-7.07803	-0.06549
C	2.52305	-4.93177	-1.19977
H	1.96924	-4.87923	-2.14935
H	3.22540	-4.08671	-1.16254
H	3.11235	-5.86128	-1.19639
C	2.37378	-4.97411	1.31400

H	3.05779	-4.11659	1.39172
H	1.71056	-4.96749	2.19223
H	2.97510	-5.89559	1.34312
C	0.62064	6.19522	-0.06961
H	0.02051	6.21644	-0.99179
H	-0.06087	6.26688	0.79114
H	1.25979	7.09024	-0.06460
C	2.32546	4.99637	1.31515
H	1.66216	4.98308	2.19323
H	3.01789	4.14562	1.39279
H	2.91770	5.92371	1.34455
C	2.47567	4.95609	-1.19856
H	3.18634	4.11798	-1.16139
H	1.92269	4.89832	-2.14831
H	3.05583	5.89133	-1.19481
C	-2.95070	-0.01774	-2.54326
H	-2.30430	0.86877	-2.64161
H	-2.29924	-0.90059	-2.64144
H	-3.65767	-0.01983	-3.38330
C	-2.91315	-0.02299	2.53222
H	-2.26107	-0.90649	2.61970
H	-2.26475	0.86286	2.62274
H	-3.60811	-0.02590	3.38222
C	-7.29856	-0.01016	0.02747
H	-7.67266	1.02723	0.02305
H	-7.71479	-0.51012	-0.85877
H	-7.70049	-0.49775	0.92704
